# Circulating Tumor Cell Detection and Capture by Photoacoustic Flow Cytometry *in Vivo* and *ex Vivo*

**DOI:** 10.3390/cancers5041691

**Published:** 2013-12-10

**Authors:** Ekaterina I. Galanzha, Vladimir P. Zharov

**Affiliations:** 1 Phillips Classic Laser and Nanomedicine Laboratories, University of Arkansas for Medical Sciences, 4301 West Markham Street, Little Rock, AR 72205, USA; E-Mail: egalangha@uams.edu; 2 Arkansas Nanomedicine Center, University of Arkansas for Medical Sciences, 4301 West Markham Street, Little Rock, AR 72205 USA

**Keywords:** *in vivo* flow cytometry, metastasis, circulating tumor cells, photoacoustic method, photothermal imaging, CTC assay *ex vivo/in vitro*

## Abstract

Despite progress in detecting circulating tumor cells (CTCs), existing assays still have low sensitivity (1–10 CTC/mL) due to the small volume of blood samples (5–10 mL). Consequently, they can miss up to 10^3^–10^4^ CTCs, resulting in the development of barely treatable metastasis. Here we analyze a new concept of *in vivo* CTC detection with enhanced sensitivity (up to 10^2^–10^3^ times) by the examination of the entire blood volume *in vivo* (5 L in adults). We focus on *in vivo* photoacoustic (PA) flow cytometry (PAFC) of CTCs using label-free or targeted detection, photoswitchable nanoparticles with ultrasharp PA resonances, magnetic trapping with fiber-magnetic-PA probes, optical clearance, real-time spectral identification, nonlinear signal amplification, and the integration with PAFC *in vitro*. We demonstrate PAFC’s capability to detect rare leukemia, squamous carcinoma, melanoma, and bulk and stem breast CTCs and its clusters in preclinical animal models in blood, lymph, bone, and cerebrospinal fluid, as well as the release of CTCs from primary tumors triggered by palpation, biopsy or surgery, increasing the risk of metastasis. CTC lifetime as a balance between intravasation and extravasation rates was in the range of 0.5–4 h depending on a CTC metastatic potential. We introduced theranostics of CTCs as an integration of nanobubble-enhanced PA diagnosis, photothermal therapy, and feedback through CTC counting. *In vivo* data were verified with *in vitro* PAFC demonstrating a higher sensitivity (1 CTC/40 mL) and throughput (up to 10 mL/min) than conventional assays. Further developments include detection of circulating cancer-associated microparticles, and super-resolution PAFC beyond the diffraction and spectral limits.

## 1. Introduction

Up to 90% of cancer deaths are related to metastasis in the distant organs due to the hematogenous and lymphatics dissemination of circulating tumor cells (CTCs) shed from the primary tumor [1,2,3,4,5,6,7,8,9,10,11,12,13,14,15,16,17,18,19,20,21,22,23,24,25,26,27]. Despite major efforts, challenges remain to treating advanced stages of disease in patients in whom distant metastases develop [6,7]. It would be extremely helpful to have an ultrasensitive blood cancer test (liquid biopsy) for early stages of metastatic diseases, when well-timed therapy is more effective. 

**Figure 1:**
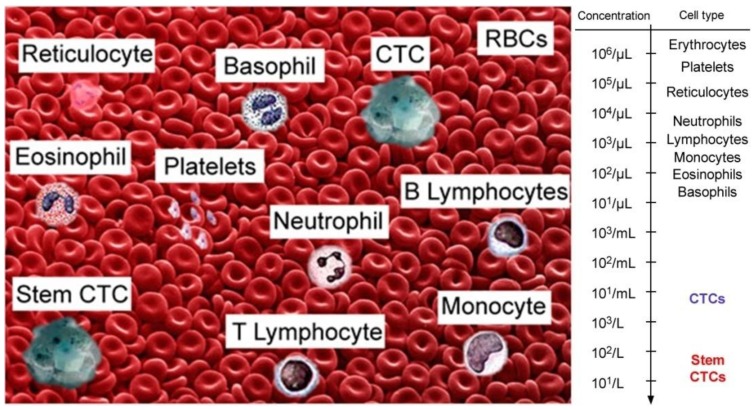
Typical blood composition and concentration levels of bulk and stem CTCs in blood cells.

**Figure 2:**
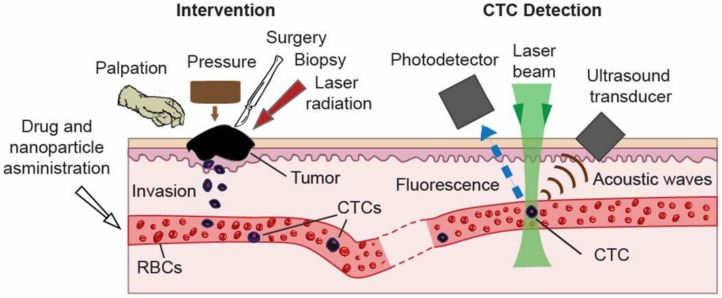
Schematic of integrated *in vivo* flow cytometry (FC). (Right) PA and fluorescence detection of CTCs with absorption or/and fluorescence properties; (Left) Natural and enforced CTC release in circulation from a primary tumor or metastasis during diagnostic and therapeutic interventions [28,29].

Comprehensive clinical studies have demonstrated the tremendous potential of using CTCs as a marker of metastatic development (the lower the CTC count, the longer the survival time), cancer recurrence, and therapeutic efficacy (~50 reviews in 2012 alone) [1,2,3,4,5]. CTCs at early disease stages are present in the bloodstream in extremely low concentrations: (≤10) CTC/mL (Figure 1). The invasion of tumor cells in the circulation may occur very early in tumor development, thus emphasizing the potential importance of sensitive detection of CTCs and circulating tumor microemboli (CTM). Moreover, some diagnostic or therapeutic procedures (e.g., palpation, biopsy, surgery, drugs) can provoke CTC and tumor product release into blood vessels at early disease stages (Figure 2, left) [28,29,30]. As recently discussed, invasive tumor cells tend to lose their epithelial antigens by the epithelial-to-mesenchymal transition (EMT) process [17]. Furthermore, non-tumor epithelial cells can also be present in blood. Thus, it appears that identification of CTCs and CTMs cannot be based on the expression of epithelial-specific transcripts or antigens only. Consequently, the technical challenge in this field consists of finding “rare” (just a few) CTCs mixed with the ~10 million white blood cells (WBCs) and 5 billion red blood cells (RBCs) in 1 mL of blood, and being able to distinguish them from epithelial, non-tumor cells and leukocytes.

To date, a variety of assays have been developed to detect CTCs in a sample of peripheral blood, including reverse transcription-polymerase chain reaction (RT-PCR), flow cytometry, optical sensors, negative selection, cell-size filtration, immune-magnetic techniques (e.g., CellSearch^®^), and microfluidic chips, among many others [8]. Combined with cell enrichment and isolation techniques, these methods provide insight on CTC concentration at different disease stage, and demonstrate clear prognostic value. However, each of these methods requires additional improvement and optimization before large-scale clinical application. The key limitation of most methods is the blood-sampling procedure. Barely treatable metastasis can already have developed at the time of the initial diagnosis with most existing CTC assays, which currently have a sensitivity of 1–10 CTCs/mL (*i.e.*, 1,000–50,000 CTCs in the entire adult blood volume of ~5 L) and are limited by the small blood sample obtained (5–10 mL). 

Other limitations of current CTC assays include: (1) labor-intensive and time-consuming procedures; (2) discontinuous CTC testing at limited, discrete time points, which does not allow real-time monitoring of CTCs in a manageable timeframe to improve patients’ chances of survival; (3) limited access to clinically relevant anatomic sites, such as the primary tumor area or sentinel lymph nodes; and (3) high false-positive and false-negative rates. In particular, some difficulties in reproducing the results of the initially broadly used RT-PCR assay are associated with differences in sample processing and the generation of false-positive signals due to contamination events, amplification of pseudogenes, and illegitimate transcription [26,27]. False-negative signals, in contrast, are related to the poor quality of source materials. Further, RT-PCR is an indirect method and cannot provide direct evidence of the presence of viable CTCs with the ability to produce metastasis. 

Most assays have also limited capability for multiplex targeting of CTCs by simultaneously using several molecular markers. Cell Search^®^ which is currently the only FDA-approved CTC assay, and several microfluidic chip-based assays (e.g., iChip) use immune-magnetic beads to isolate CTCs expressing epithelial cell adhesion molecule (EpCAM) [8,12]. Isolated CTCs are further distinguished by being labeled with cytokeratin, DAPI, and use antibody (Ab) against CD45 as a marker of WBCs. To ensure the presence and isolation of rare CTCs, relatively large blood volumes are required, typically 10–20 mL, and even 50 mL [8]. Modern microfluidic systems process this volume at a slow flow rate, in the range of 1–2 mL/h, and require up to 25 h (theoretically 6 h with a recent 8 mL/h system [18]). Fluorescence imaging after processing allows analysis of cell morphology, integrity, and viability, as well as the presence of a nucleus to differentiate CTCs from RBCs and WBC fragments due to necrotic and apoptotic phenomena in blood flow. Existing CTC assays cannot provide high-speed dynamic detection and imaging, thus making CTC analysis using even single markers time consuming. Moreover, many CTC assays require multiple processing and measuring steps, including CTC labeling, immune-magnetic enrichment, RBC and WBC separation, additional sorting, and a second fluorescent labeling resulting in a substantial loss in the number of CTCs (up to 60%–95%) [1,2,3,4,5]. 

Conventional flow cytometry (FC) *in vitro*, currently the most powerful analytic tool, can potentially provide multiplex (up to 6–8 markers) detection of CTCs using multicolor probes [31,32]. In this technology, labeled cells in fast (a few m/s) flow with a high cell rate (up to 10^5^ cells/s) are accurately positioned by hydrodynamical or acoustical focusing into a single file (5–10 µm in diameter) that passes through tightly focused laser beams used to detect scattering and fluorescent light from individual cells, as well as providing dynamic imaging at a rate of 10^2^–10^3^ cell/s. This tool has been broadly used for accurate, multiparameter quantification of individual cells including studies of cell functional states, morphology, composition, proliferation, and protein expression, and for immunophenotyping of a variety of specimens, including whole blood, bone marrow, serous cavity fluids, cerebrospinal fluid, urine, and solid tissues. Applications in hematology include DNA content analysis, leukemia and lymphoma phenotyping, immunological monitoring of HIV-infected individuals, and assessment of structural and functional properties of erythrocytes, leukocytes, and platelets. For the analysis or rare CTCs, however, modern FC is limited by low throughput as other CTC assays (above). 

In addition, invasive extraction of blood from a living organism may alter CTC parameters (e.g., signaling, epigenetic states, metabolic activities, or morphology) and prevent the long-term study of CTC properties (e.g., CTC-blood cell interactions, aggregation, rolling, or adhesion) in their natural biological environment. These problems can be solved by assessing a significantly larger volume of blood up to a patient’s entire blood volume with *in vivo* FC, whose principles were proposed in 2004 simultaneously by Zharov and other groups [33,34,35,36,37,38,39,40,41,42,43,44,45,46,47,48,49].

## 2. *In Vivo* Flow Cytometry

*In vivo* FC preferentially uses fluorescence (*i.e.*, as in conventional FC) [38,39,40,41,42,46,47,48,49] and photothermal (PT) and photoacoustic (PA) detection methods [28,33,34,35,36,37], and, recently the combination of these methods [28,29,49]. A brief history and the features and challenges of this next generation of FC have been described in reviews and book chapters ([33,34,43,44,45,46,47,48] and references there) with a focus on early work in this field and broad applications, including circulating RBCs and WBCs in different functional states (e.g., normal, apoptotic), infections (*E. coli* and *S. aureus*), sickle cells, blood rheology, and pharmacokinetics of nanoparticles (NPs), drug carriers, and dyes and other contrast agents. Here, we summarize recent advances of *in vivo* FC platform with a focus on detecting CTCs with PAFC methods. 

### 2.1. General Schematics of *in Vivo* Flow Cytometry

The individual cells in various types of bioflows (e.g., blood, lymph, or cerebrospinal fluid) are irradiated with one or several laser beams at different wavelengths (Figure 2, right). Laser-induced optical effects in individual cells, such as absorption, PT and PA phenomena, fluorescence, and elastic and inelastic (Raman) scattering are detected with optical or non-optical (e.g., ultrasound) detectors, depending on the method used. Counting of each cell *in vivo* can be achieved in a small capillary with diameter of 6–10 µm, where 5–6 µm RBCs flow one by one [28]. Due to the slow flow velocity (0.1–0.5 mm/s), cell rate is extremely low (<30 RBCs/s). Moreover, a majority of the cells of interest such as CTCs or WBCs with a typical diameter of 12–25 μm and 8–9 μm, respectively, cannot enter into the capillary. In larger vessels, many cells are simultaneously present in the irradiated volume (e.g., hundreds in 50–100 µm-diameter blood vessels) with multiple-file flow and unstable positions in the vessel cross-section. These conditions make it extremely difficult to distinguish and count each cell in the detection volume. However, *in vivo* FC can count individual and rare CTCs possessing intrinsic optical properties (e.g., absorption) or labeled with exogenous labels (e.g., fluorescence or strongly absorbing NPs) against the background of the many blood cells in the detection volume. 

*In vivo* FC, with fluorescence detection of CTCs, can be built on the basis of conventional and confocal microscope schematics, single-, two-, or multiphoton excitation, and standard fluorescent labels, as in conventional FC *in vitro* [38,39,46]. In the confocal scheme, fluorescent signals from the cell population of interest are recorded as the cells pass through a slit of a continuous-wave (CW) laser (e.g., He-Ne) light focused across 20–50 µm mouse ear blood vessels. Emitted fluorescence is collected by the microscope objectives and directed through a dichroic splitter and mirrors to photomultiplier tubes. Compared to single-photon fluorescence FC, multiphoton fluorescence technique can increase the depth of light penetration in microvessels located deeper in tissue (a few hundred μm) and reduce out-of-focus photodamage. However, this technique typically uses focused circle laser beams that may lead to missing cells flowing in relatively large vessels outside the irradiated zone. Recent advances include the use of two-color schematics and fiber-based laser delivery [42,47]. *In vivo* fluorescence flow cytometry (FFC) was successfully used for *in vivo* detection of labeled CTCs in animal models [46,47,48,49]. Nevertheless, the use of fluorescent labeling raises concerns due to the cytotoxicity of fluorescent tags and their usefulness for assessing only superficial microvessels (≤50–100 µm) with slow flow velocities (1–5 mm/s) due to the influence of a strong autofluorescent background. As a result, counting of CTCs with FFC even in an animal model—and especially with respect to translation for use in humans—is a technical challenge, and an optimal solution has not yet been found. 

### 2.2. Animal Models

**Figure 3:**
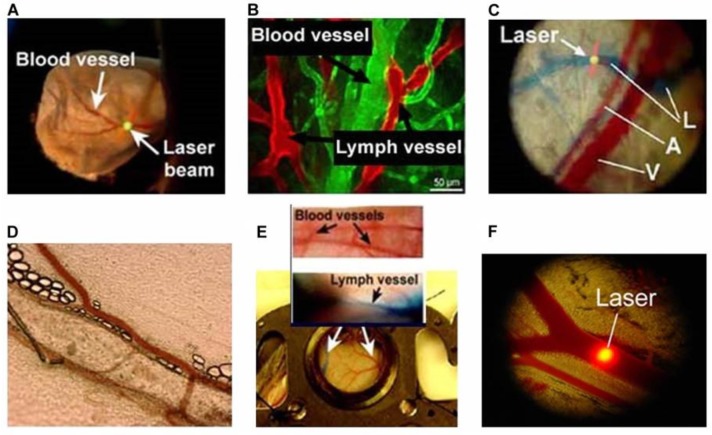
(**A**) Photo of nude mouse ear; (**B**) Fluorescence images of mouse ear vasculature; (**C**) Optical (transmission) image of mouse ear: A, arterial; V, vein; L, Evans blue-labeled lymph vessels; (**D**) Optical images of lymph and blood microvessels of rat mesentery; (**E**) Window chamber in the skin of a murine model; (**F**) Circular laser beam on a blood vessel [50,51,52].

Most studies *in vivo* FC have been performed in thin (250–300 μm), relatively transparent mouse ear in which both veins and arteries are well distinguished (Figure 3A–C). These ear blood vessels, lying 30–100 μm deep, have diameters in the range of 30–50 μm and blood velocities of 3–7 mm/s. Many experiments have also been performed with rat and mouse mesentery, which has an almost ideal vascular and surrounding tissue structure for *in vivo* FC because it consists of very thin (7–15 µm) transparent connective tissue with a single layer of blood and lymph microvessels (Figure 3D). PAFC was also applied in 200–300 μm-diameter blood vessels in the abdominal area of the nude mouse at a depth of 0.3–0.5 mm with and without a window chamber (Figure 3E,F) on the aorta (0.9–1 mm) at a depth of 2–4 mm [28]. The animals were anesthetized by a standardized procedure and placed on a heated microscope stage with the ear spread flat over a glass slide. The ultrasound transducer was acoustically connected to the mouse’s ear with warm water or topically applied conventional ultrasound gel. 

To produce metastatic disease, the mice were inoculated with various tumor cell lines through intravenous injection into either intracardiac or tail vessels. The mice were monitored by PAFC immediately after the injection to control the clearance rate (usually a few hours) to make sure that there was no metastatic disease. The appearance of “secondary” CTCs typically occurred 1–2 weeks after injection, depending on the metastatic potential of the injected tumor cells, and was a sign of metastatic disease. Development of overt metastases at an early, latent stage was controlled with an IVIS imaging system (Caliper Life Sciences, Inc., Hopkinton, MA, USA). The selected mice were monitored with *in vivo* FC over 4–8 weeks, depending on the tumor model, to estimate the overall process of metastatic disease. 

To create a primary tumor in different locations, mice were inoculated with tumor cells either in the ear, skin, or mammary pad. Then the mice were examined weekly by PAFC, FFC, or intravital fluorescence imaging with cells expressing green (or another color) fluorescent protein (GFP) to control primary tumor progression (e.g., size, vascularity) and whole-body IVIS imaging to define the appearance of overt metastases. At the end of the *in vivo* studies, blood and multiple organs (e.g., liver, lymph nodes, kidneys, lungs, brain) were usually extracted for examination with PAFC *in vitro*, PT and PA microscopy, and conventional histology.

## 3. PA Flow Cytometry (PAFC)

### 3.1. Principle of PAFC

PA methods are based on nonradiative conversion of absorbed light energy into heat (PT effect) accompanied by acoustic waves (PA effect) [53,54,55,56,57,58,59]. Specifically, absorption of laser radiation by a single cell leads to a temperature increase in endogenous and exogenous structures (e.g., NPs). The temperature distribution is transformed into a refraction distribution that can be detected with PT methods using a second probe beam through a change in its parameters (e.g., phase or defocusing effects) recorded with phase-contrast, heterodyne, and thermal-lens techniques [60,61,62,63,64,65,66,67]. The thermal expansion of heated zones leads to the generation of acoustic waves detected with PA methods [53,54,55,56,57,58,59]. PT and PA methods, analogous to conventional absorption spectroscopy, provide information on nonfluorescent cellular absorption associated with intrinsic chromophores and pigments (e.g., cytochromes, hemoglobin, carotenoids, melanin, DNA/RNA, water, or lipids). Strongly absorbing, low-toxicity NPs functionalized with small proteins or antibody fragments (e.g., Fab) can be used as high-contrast PT and PA agents for molecular targeting of CTC markers that significantly increase both the specificity and the sensitivity (10–10^3^-fold of PA and PT methods [28,68]. PT techniques, in particular PT microscopy, offer the highest absorption sensitivity [66,67] (1–2 orders of magnitude better than PA microscopy [PAM] and 4–5 orders better than absorption spectroscopy), providing the unprecedented capability for label-free detection of single molecules [69] and single ~1 nm gold NPs *in vitro* [70]. In contrast, PAM can only detect NPs larger than 30–60 nm against a cellular background. However, PA techniques offer the advantage of being able to image more deeply (up to 5–7 cm) in tissue *in vivo* [56]. In addition, the high level of sensitivity enables PT and PA detection and imaging of cells noninvasively (*i.e*., with a short-term temperature elevation ≤0.1–1 °C). The PT and PA methods complement each other because PT techniques provide better sensitivity for studying thin, relatively transparent structures like animal ear or mesentery, while PA methods demonstrate an advantage in assessing deep vessels. In addition, PA methods (1) are not sensitive to light scattering and autofluorescence, (2) can provide label-free detection of melanoma CTCs by using intrinsic melanin as a PA contrast agent, and (3) require a low laser energy within the laser safety standards to generate detectable PA signals. 

To generate PA signals in each moving CTC, a high-pulse-repletion-rate laser is used in PAFC. Each flowing CTC will be exposed at a laser pulse rate f_r_ ≥ V_F_/d, where V_F_ is blood flow velocity and d is the width of laser beam or acoustic resolution [28]. In a 1–2 mm-diameter human vein, V_F_ is ~5–15 cm/s and d = 50–100 μm with acoustic-resolution PAFC (see section below) and hence f_r_ ≥ 0.5–2 kHz. In animals with a slower flow velocity, a laser pulse rate can be lower. Increasing the pulse rate improves the signal-to-noise ratio (SNR) by averaging PA signals from each exposed CTC. The pulse width t_P_ must satisfy acoustic confinement, providing the generation of the maximum PA signal [28]: t_P_ ≤ τ_A_ = 2R/c_s_, where τ_A_ is the transit time of the acoustic wave traveling through an absorbing target with radius of R, and c_s_—is the speed of sound. For typical conditions, t_P_ ≤ 10 ns and t_P_ ≤ 0.5 ns for R_CTC_ = 8 μm (size of a whole, strongly pigmented melanoma cell) and R_M_ = 0.4 μm (approximate size of one melanosome or NP cluster,) respectively. 

In conventional, positive-contrast *in vivo* PAFC [71,72,73,74,75,76,77,78,79,80,81,82,83,84,85,86,87,88,89,90,91,92,93,94,95,96,97,98,99,100,101,102,103,104,105], the SNR is determined by the ratio of the flash (transient) PA signal from single CTCs to the superposition background PA signals from RBCs in the detection volume, and to a noise of different origins (e.g., electronic, acoustic, fluctuation in RBC number, or instability of laser energy [typically 3%–5%]). In particular, we verified in multiple studies using the *in vivo* mouse model and *ex vivo* human blood spiked with melanoma cells (e.g., B16F10, HTB-65, CI81, or SK-ML-7) that PAFC in the optimal NIR range (e.g., at 820 nm or 1,064 nm) can detect single pigmented melanoma CTCs in the presence of 100–300 RBCs, which is in line with coefficients of absorption for blood and melanin (see details in ref. [82]). As mentioned above, to enhance PA signals from non-pigmented CTCs, they are molecularly targeted by strongly absorbing NPs [81,85]. 

### 3.2. General Schematics of PAFC

The PAFC setup was equipped with tunable optical parametric oscillators (OPOs) having a spectral range of 420–2,200 nm, a pulse width of 5–8 ns, pulse-repetition rates of 10 and 100 Hz, pulse energy of 2 mJ, and four high-pulse-repetition-rate lasers with following parameters: main wavelength: 532, 671, 820, and 1,064 nm; pulse width: 5–10 ns; pulse rate: 1–500 kHz; pulse energy: 50–100 µJ (see detailed parameters in refs. [28]). Laser radiation can be delivered to biotissues by using either a microscope schematic with a cylindrical lens to create the desired linear beam shapes (e.g., from 5 × 50 µm to 25 × 150 µm), or a fiber with a miniature tip and cylindrical optics. PA signals are detected by various unfocused and focused ultrasound transducers (see details in [28]). In particular, these include a cylindrical transducer (frequency, 20 MHz; focal length, 12.5 mm; model V316-SM; Olympus NDT, Inc., Waltham, MA, USA) and a customized cylindrical transducer (frequency, 30–35 MHz; focal length, 4–8 mm; lateral resolution, 45–60 µm) with a hole for an optical fiber. PA signals with a typical bipolar shape and a duration of 0.1–0.5 µse are then amplified (model 5662: bandwidth, 50 kHz–5 MHz; gain, 54 dB; model 5678: bandwidth, 40 MHz; gain, 60 dB; both from Olympus Panametrics-NDT). 

This setup has been integrated with PT, transmission digital microscopy (TDM), and fluorescence imaging modules using CCD (DP42, Olympus America Corporate, Center Valley, PA, USA) and a high-speed CMOS camera (model MV-D1024-160-CL8; up to 10,000 frames per second [fps]) [28,74]. In PT thermal lens mode, pump laser (OPO)-induced temperature-dependent variation of the refractive index in single cells causes defocusing of a colinear CW laser probe beam (633 nm, ~1 mW) that reduces the beam’s center intensity as detected by a photodiode through a pinhole and recorded as the integrated PT signal [66,67]. The linear PT signal represents a rapidly occurring peak associated with rapid (ps-ns scale) cell heating with a slower (µs scale) tail corresponding to cell cooling. In non-linear mode, overheating of localized absorbing zones is accompanied by short-lasting (e.g., 10–500 ns) nanobubbles manifested by sharp negative PT peaks due to strong refraction and scattering effects. In PT imaging mode, OPO-induced variations of the refractive index are visualized with a multiplex thermal lens scheme using a second collinear laser probe pulse at 639 or 671 nm with a tunable (0–10 µs) delay between pump and probe pulses [63,66]. Formation of a PT image requires just one laser pulse having a relatively broad beam diameter (10–25 µm) covering each entire cell or spatial sample (or laser) scanning [66]. 

### 3.3. PAFC with Optical and Acoustic Resolution

**Figure 4:**
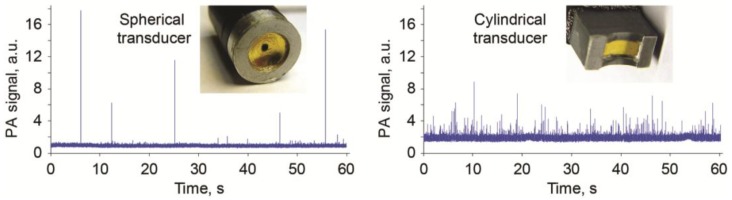
PA signal traces from melanoma cells (B16F10) in mouse blood with focused spherical (left) and cylindrical (right) ultrasound transducers with focal lengths of 6 mm and lateral resolution of ~60 µm. These data were obtained by Y. Menyaev.

With respect to PAFC’s optical and acoustic schematics, PAFC can be defined as optical-resolution PAFC (OR-PAFC) and acoustic-resolution PAFC (AR-PAFC) [28,83,102]. In OR-PAFC, resolution is determined by optical parameters, in particular, the minimal width of a focused linear laser beam. Due to strong light scattering in tissue, high spatial resolution at the level of 5–10 µm in OR-PAFC can be achieved in superficial 30–50 µm-diameter vessels only at the shallow depth of 0.1–0.2 mm. In AR-PAFC, in deeper tissue with stronger light scattering, the resolution depends on ultrasound focal parameters (e.g., 20–100 µm at a frequency of 10–50 MHz, respectively). A comparison of focused cylindrical with spherical transducers conducted during PA assessment of mouse blood spiked with a low concentration of a melanoma cell line (B16F10) flowing in an 0.8 mm-diameter tube showed that a spherical transducer provided a higher SNR (3–5-fold) than cylindrical transducers (Figure 4, right), although with fewer CTC-associated PA peaks (Figure 4, left). This can be explained by the smaller detection volume and hence the lower number of RBCs producing background signals with the use of a spherical transducer. However, CTCs flowing outside this volume were missed. This finding suggests that a focused cylindrical ultrasound transducer is better suited for PA detection of CTCs because it provides a minimal detection volume due to high lateral resolution that can simultaneous assess the entire cross section of a vessel. However, its sensitivity a little lower than a spherical transducer due to the higher blood background signals. 

### 3.4. Nonlinear PAFC for CTC Contrast Enhancement

As mentioned above, PA detection of single CTCs *in vivo* can be limited by absorption by the blood background, which is determined by the number of RBCs in the detection volume. In our early work [53,54], we proposed various approaches to reduce the influence of background absorption, including: (1) generation of second-harmonic PA signals from saturated absorption in targets in the presence of a linear background; (2) multiphoton absorption; (3) two-beam excitation with different wavelengths and modulation frequencies, (4) discrimination of targets with different temperature-dependent absorption and relaxation times; and (5) changes in blood oxygenation, osmolarity, and hematocrit within physiological norms (see details and refs. in [82,102]). 

**Figure 5:**
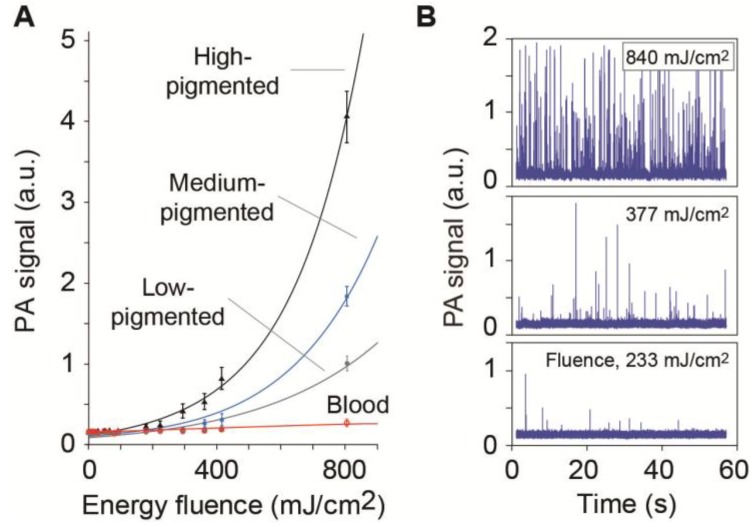
Nonlinear PA signal amplification at 820 nm in melanoma cells (B16F10) with different pigmentation under static (**A**) and flow (**B**) conditions as a function of the laser energy fluences [102].

Another of our approach to provide nonlinear PA signal amplification from single targets against linear absorption background is based on differences in the dependence of the PA signal on the laser energy from targets and background. Among different nonlinear phenomena, we focused on laser generation of nanobubbles as more efficient (5–50-fold) PA signal amplifiers from strongly absorbing, highly localized targets in the presence of the spatially homogenous absorption background generating only linear signals with no nanobubble formation. This approach was first used to enhance the contrast of PT and PA imaging of melanoma cells, NPs, and NP clusters in histological, cancer-related samples, with simultaneous spectral burning of background absorption with the use of different laser wavelengths coinciding with the maximum absorption of the CTCs or the background [104]. In view of ability of laser-induced nanobubbles and microbubbles to enhance PA signals and simultaneously mechanically destroy CTCs, we recently applied this approach for the theranostics of individual CTCs using the nonlinear PAFC for label-free detection of circulating melanoma cells (B16F10) against the blood background *in vitro* and *in vivo* [82,94,95,102]. The thresholds of nanobubble generation demonstrated high sensitivity to melanin or NP clustering (e.g., the larger the nanocluster, the lower the threshold) and were used to control their clustering in tumor cells [82]. A nanobubble-associated, nonlinear PAFC was tested initially for label-free detection of single melanoma cells (B16F10) against the blood background *in vitro* and *in vivo* [102]. Specifically, at a low laser energy level, the PA signal from a single weakly pigmented melanoma cell was below that of the blood background as PA signals from individual RBCs are superposed in the detection volume. At higher energy levels, nonlinearly amplified PA signals from overheated melanin nanoclusters in melanoma cells became detectable above the linear blood background. In particular, we performed detailed measurements of PA signals from melanoma cells with different degrees of pigmentation in blood as a function of laser fluences. We observed significant (10–30-fold) signal amplification from these cells, as compared to linear effects from RBCs with relatively spatially homogeneous hemoglobin distribution (Figure 5A), that led to detecting more CTC-associated PA peaks at higher laser fluences (Figure 5B). Thus, nonlinear nanobubble-based PAFC provides a new opportunity to significantly increase PA contrast of single nanoparticles, cells, viruses, and bacteria in complex biological environments. 

### 3.5. Real-Time Spectral Identification of CTCs

To make the setup more adaptable for studying CTCs and labels with different absorption spectra, we used a spectrally tunable OPO and high-pulse-repetition-rate lasers with different wavelengths [28]. The lifetime of CTCs in the detection volume is short, in the range of 0.1–2 ms, which makes it extremely difficult for spectral identification of fast moving CTCs. In the past, with a similar problem associated with chromatography using PA detectors for spectral identification of moving, spatially separated compounds in chromatographic columns, we developed (1) a fast spectrum-scanning laser; (2) multiplex spectral detection by simultaneous irradiation of moving objects with several laser beams at different wavelengths, modulated at different acoustic frequencies (analogous to discrete PA Fourier spectrometry), and (3) fast switching between two laser wavelengths (*i.e.*, laser discrete frequency modulation) ([53,54,75] and references there). Recently, we used real-time multicolor PA spectroscopy at discrete wavelengths with laser pulses at different wavelengths and pulse delays in combination with time-resolved detection of PA signals [81,82,105]. We selected a pulse-repetition rate of 10–30 kHz for all lasers and a delay between laser pulses in the range of 5–25 μs, depending on the number of wavelengths. Laser pulses were triggered by a digital delay/pulse generator (DG645; Stanford Research Systems, Inc., Sunnyvale, CA, USA) for time and color coding. Each laser was driven by an independent triggering channel at the selected pulse rate, with delays between consecutive channels. Thus, the delay between laser pulses with different wavelengths provided time-color coding for time-resolved detection of different “color” PA signals with a single ultrasound transducer. Parallel linear laser beams (4–10 µm × 0.1–1 mm) of different wavelengths can be either overlapped in the sample plane or separated by adjustable gaps. The PA signals were recorded with a high-speed analog-to-digital converter board (e.g., PCI-5152, National Instruments Corporation, Austin, TX, USA; ATS9350, Alazar Technologies Inc., Pointe-Claire, Canada) and, after acquisition and averaging (e.g., 10–50 PA signals from each CTC), were presented as signal traces in which amplitudes, peak widths, and coincidence in different traces were analyzed with customized software. This made it possible in “real-time” to analyze PA signals from the same CTCs at different laser wavelengths. Similar techniques have recently been applied for measuring RBC oxygenation at two wavelengths [106].

Nevertheless, the spectral capability of PAFC can be limited to wide NIR spectral bands (50–200 nm in width) of chromophores and NPs, especially plasmonic NPs. Recently, we discovered that nanobubble-induced ultrasharp nonlinear PA resonances in various absorbing structures could be used to enhance the multicolor PAFC’s capability through dramatic sharpening of the spectral bands to 1–5 nm wide [28,96,103,107]. The mechanism of these resonances is associated with laser-induced non-linear amplification (10–100-fold) of PA signals near the center of the absorption bands. A tuning of the laser wavelength toward the absorption center leads to increased absorption of energy, raising the temperature above the nanobubble-formation threshold and accompanied by significant non-linear signal amplification. As a result, spectrally dependent signal amplification leads to the sharpening of PA resonances near the center of the absorption peaks at an optimal laser energy. High laser energy can lead to PT or photochemical modification of absorbing structures during the laser pulse, and even at the beginning of the pulse. In turn, this is accompanied by decreased absorption and signal inhibition that leads to formation of spectral holes and spectral splitting of originally single peaks [96,108], together with off-center ultrasharp resonances, particularly red-shifted resonances in plasmonic NPs [28,107]. These phenomena are relatively universal and applicable to various absorbing nanostructures. In particular, we demonstrated dynamic spectral sharpening in different single and clustered NPs (e.g., gold nanospheres, quantum dots [QDs], golden carbon nanotubes [GNTs], gold nanoshells (GNSs), gold nanorods [GNRs], and magnetic NPs [MNPs]), cellular chromophores (e.g., melanin, hemoglobin, cytochromes), and dyes (e.g., fluorescein isothiocyanate [FITC] and indocyanine green [ICG]) [94,103,107]. More profound sharpening (up to 0.8–1 nm wide) was observed in plasmonic NPs (GNRs and GNTs), while typical widths of PT and PA resonances for other objects were in the range of 2–10 nm. As mentioned above, nonlinear, ultrasharp PA spectral resonances were accompanied by significant amplification of PA signals that led to dramatic increases in both the specificity and the sensitivity of PAFC and enhanced the efficiency of PT theranostics [95]. 

### 3.6. Optical Clearance in PAFC

**Figure 6:**
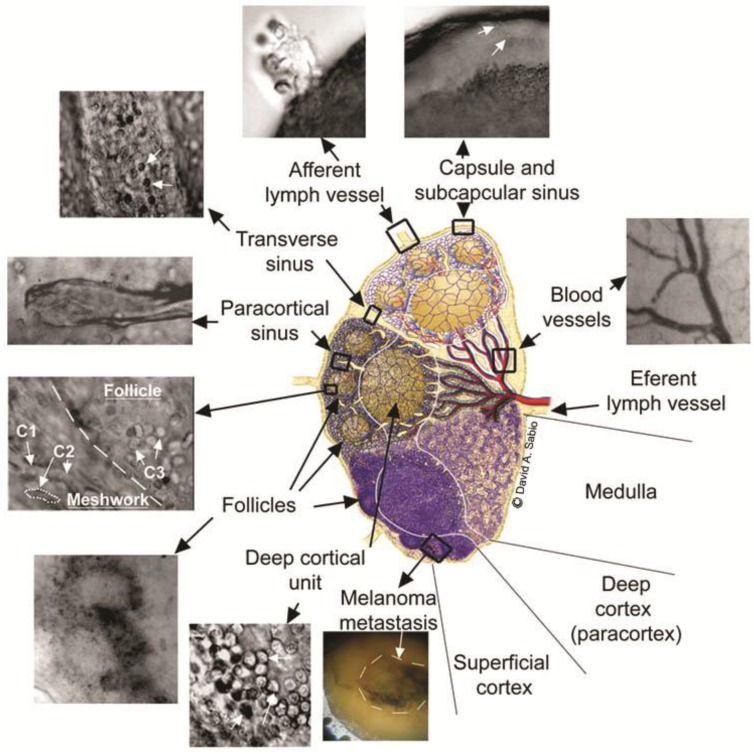
Optical imaging of the microanatomy of a fresh lymph node *ex vivo* obtained with optical clearance using 80% glycerol. The central schematic shows a midsagittal section of a lymph node containing three lymphoid lobules with the basic anatomical and functional unit of the lymph node. Top and middle lobules: microanatomical schematics of lobular compartments (superficial cortex, deep cortex, and medulla) without (top) and with (middle) reticular meshwork. Bottom lobule: a lobule of a mouse lymph node as it appears in conventional histological section. C1 shows basophilic lymphocytes; C2 shows elongated fibroblastic reticular cells; and C3 shows B lymphocytes and follicular dendritic cells [83].

**Figure 7:**
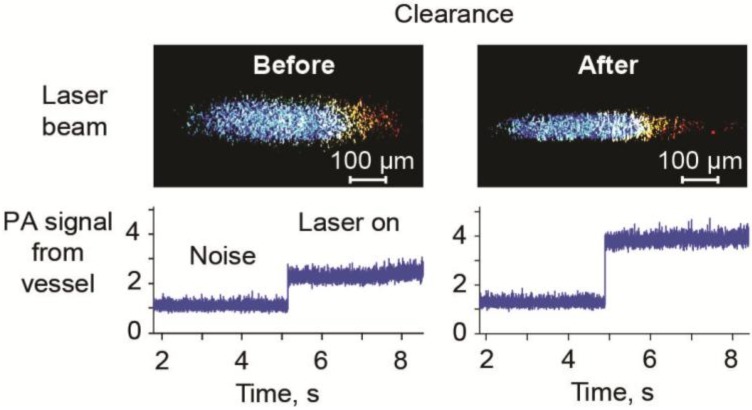
Laser beam after passing through 0.9 mm-thick layer of fresh mouse skin (top) and PA signals from a 1 mm-diameter, 1.3 mm vein (bottom) before (left) and after (right) optical clearance [70].

To reduce the light scattering, optical tissue clearance has been used in optical imaging [109]. In 2006, we proposed, for the first time, using optical clearance to enhance the PA method, particularly PAFC’s capability to assess deep vessels, and image sentinel lymph nodes (SLNs) [71,74,83]. Specifically, to decrease beam blurring due to light scattering in skin and lymph node tissue, we compared clearance effects by administration for 5 min of 40% glucose, 100% dimethyl sulfoxide, and 80% glycerol as hyperosmotic “clearing” agents. Comparisons of optical images with different agents and phosphate-buffered saline (control) revealed that glycerol offered the maximum optical clearance. Treatment of SLNs with 80% glycerol permitted the label-free imaging of a fresh node at the cellular level, including localization of immune-related, metastatic, and other cells (e.g., lymphocytes, macrophages, dendritic cells, and melanoma cells), as well as surrounding microstructures (e.g., afferent lymph vessel, subcapsular and transverse sinuses, medulla, reticular meshwork, follicles, and veins) (Figure 6). Later, a similar approach was explored for PA microscopy [110]. In our recent study [70], we optimized rapid (10–20 min) optical clearance of skin to minimize light scattering and thus increase PAFC’s optical resolution and sensitivity by sequent skin microdermabrasion, glycerol administration, and sonophoresis. A linear laser beam of 6 µm × 600 µm, after propagation through fresh 0.9 mm-thick mouse skin, was attenuated 2.5–3-fold and blurred into an ellipsoidal shape ~90 µm wide (Figure 7, top, left). After optical clearance over 10–20 min, a partial reduction in the influence of scattering light resulted in a 1.6-fold decrease in laser spot blurring (Figure 7, top, right). As a result, the PA peak rate, obtained with a focused cylindrical transducer, from B16F10 melanoma cells in mouse blood flow in a 0.8 mm-diameter capillary tube covered by a layer of mouse skin increased 1.7-fold due to an increased laser energy fluence [70]. We then applied a similar procedure in healthy human volunteers in accordance with a protocol approved by the UAMS Institutional Review Board in 2013 (March). PA signals were acquired from dorsum of the left hand before and after optical clearance. As a result, these procedures eventually resulted in a ~2-fold increase in PA signals from a 1 mm-diameter, 1.3 mm-deep vein (Figure 7, bottom). These results suggest that optical clearance can improve the detection capability of PAFC. Monitoring of human skin and blood circulation has shown no side effects after 2 months of observation; however this important issue requires additional study.

### 3.7. Minimally Invasive PAFC

**Figure 8:**
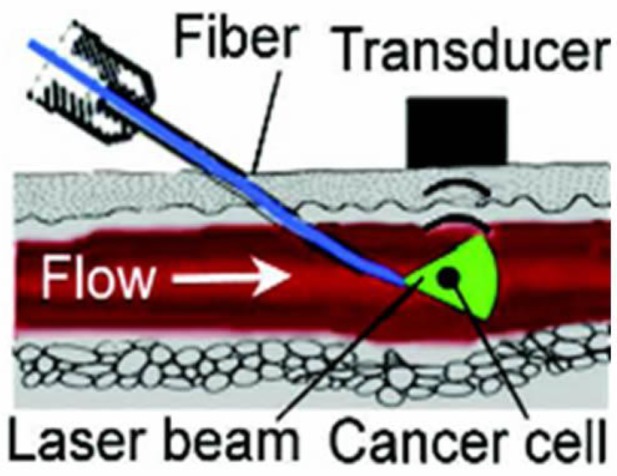
Minimally invasive fiber delivery of laser radiation into blood vessels with detection of acoustic waves by an ultrasound transducer on the skin surface.

Minimally invasive delivery of laser radiation (as proposed in [76]) through 100 µm-diameter quartz fibers in a tiny needle showed PAFC’s capability to detect melanoma CTCs in 300 µm-diameter mouse abdominal skin vessels (Figure 8) [82]. This schematic can be used to distinguish between rare individual CTCs with relatively large distances between them (e.g., >0.1–1 mm at a concentration of <10–10^2^ CTCs/mL) that makes the requirement to have a high resolution of PAFC is less strict. In testing this scheme in our animal models, we have used a laser fluence at the fiber tip in the range of 50–100 mJ/cm^2^, which is within the laser safety standard of 100 mJ/cm^2^ at a laser wavelength of 1,064 nm and a pulse rate of 10 Hz. However, as mentioned above, this safety issue require further careful verification using fibers with different diameters. 

### 3.8. Labeling *in Vivo*

The great advantage of *in vivo* FC is the possibility to detect cells without labeling, particularly by using high PA contrast of pigmented melanoma CTCs above the background signal of blood [82]. Cells with low endogenous absorption can be labeled directly in the bloodstream through intravenous injection of functionalized NPs [49,81,94]. Depending on cell and tag properties, the labeling procedure in a mouse model takes from 10–20 min to 1 h. Labeling specificity is provided through the selection of molecular markers that are highly expressed in targeted CTCs but are almost absent in normal blood and endothelial cells. High labeling efficiency is associated with frequent label-CTC collisions in partly turbulent blood flow. In accordance with our modeling, injection of 10^10^ NPs into a mouse with a blood volume of ~2 mL provides, on average, ~10^3^ NP-CTC collisions per minute, with expected differences in the velocities of NPs and CTCs of ≤1 mm/s and shear stress of ≤0.5 dyn/cm^2^, while their absolute velocities may be 5–10 mm/s. PA signals from CTCs targeted typically with 500–5,000 NPs per CTC cell are much higher than PA background signals from RBCs, unbound NPs (typically numbering 1–10 in the detection volume), or from NPs non-specifically bound to normal blood cells (e.g., macrophages). NP clustering around naturally densely packed cancer markers can lead to more significant enhancement in PA signals (at least 5–10-fold) and a red-shift effect in the absorption of coupled NPs in clusters, both of which serve as indicators of successful cell targeting. 

### 3.9. Combination of PA, PT and Fluorescence Methods

The use of a single technique limits the range of detectable cells with different optical properties. To increase the number of detectable cells and probes, conventional FC integrates fluorescence and scattering detection methods. In 2005, we proposed the integration of PT, PA, fluorescence, and scattering detection methods for *in vivo* FC [69,70,73]. PAFC was first combined with PT flow cytometry [69,71]. An advantage of PAFC is its backward mode (*i.e.*, laser and transducer are on one side), while PTFC has better sensitivity in transillumination mode. As mentioned above, PT and PA methods beneficially supplement each other and, in combination, provide a very powerful diagnostic and therapeutic tool. For example, non-invasive PA diagnostics can be integrated with PT killing of CTCs (PA-PT theranostics) [94]. 

Recently, we integrated PAFC and fluorescence FC (FFC), using both pulsed and CW lasers as traditional sources for the generation of PA and fluorescent signals, respectively [29,49]. We used the pulsed lasers only for the simultaneous generation of signals, which simplified the PAFC + FFC schematic (PAFFC) and provided time-resolved discrimination of objects with different lifetimes (e.g., QDs with short fluorescence compared to blood with longer background autofluorescence).

**Figure 9:**
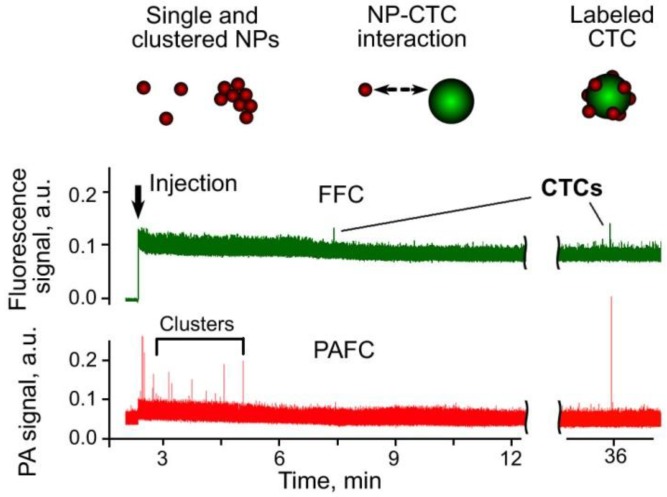
*In vivo* multimodal PA (red trace) and fluorescence (green trace) detection of CTCs (C8161-GFP) in a tumor-bearing mouse model with functionalized MNPs and GFP as PA and fluorescent contrast agents, respectively. Black arrow marks the moment of NP injection. Wavelength and energy fluence/intensity for PAFC and FFC: 820 nm and 50 mJ/cm^2^ and 488 nm and 80 W/cm^2^, respectively.

We used dual PA-fluorescence detection to study the interaction between CTCs expressing GFP and absorbing NPs [49]. We selected the C8161-GFP melanoma cell line, which provided positive fluorescent contrast. The 50 nm-diameter MNPs conjugated to anti-melanoma-associated chondroitin sulfate proteoglycan (MCSP) antibodies were used as PA molecular contrast agents to target MCSP receptors at the melanoma cell surface. Using conventional FC, we estimated that 51% of the C8161-GFP cells expressed MCSP. In control mice (*i.e.*, without tumor), PA detection at 820 nm after injection of 50 nm-diameter anti-MCSP MNPs (10^11^ NPs/mL; 10 µL of solution) revealed a few PA signals exceeding the blood background during first 3–5 min after injection. These signals were associated with small NP clusters rapidly cleared from the blood. Next, PAFFC was used to monitor the interaction of NPs and CTCs in the blood of a tumor-bearing mouse on day 20 after subcutaneous inoculation of C8161-GFP cells. FFC with a 488 nm-wavelength CW laser showed the presence of CTCs in the circulation at a rate of ~10 CTCs/h in the 50 µm-diameter ear artery (Figure 9, top). After injection of NPs, we immediately observed several PA signals associated with small NP clusters (Figure 9, bottom) that had no matches in the fluorescence trace. PA signals appeared again ~30 min after injection and matched the fluorescent data. Matching of PA and fluorescence signals confirmed successful molecular targeting of CTCs by the functionalized anti-MSCP MNPs and decreased the probability of detecting false-positive signals [63,87]. 

### 3.10. Photoswitchable PAFC

Photoswitchable fluorescent proteins (PFPs) that change their emission color in response to light have led to breakthroughs in studying static cells ([107] and references there). However, use of this technique for dynamic tracking of cells *in vivo* is challenging. Moreover, conventional photoswitching is not applicable to weakly fluorescent proteins. As an alternative, PA imaging and particularly PAFC have demonstrated tremendous potential for the study of non-fluorescent structures in the visible and NIR ranges. However, little progress has been made in the development of switchable PA probes with controllable spectral shifts in absorption. Recently, we introduced the concept of switchable PT and PA probes using conventional PFPs with conventional photoswitching mechanism as wells as plasmonic NPs with new in PT-based switching mechanisms [107]. To test this concept, we demonstrated reversible magnetic–PT switching of conventional and gold-coated MNPs in cancer cells and PT switching of plasmonic resonances in GNRs targeting circulating cells *in vivo.* We have shown that the new generation of PT-switchable probes can open a new avenue in the dynamic tracking of CTCs that provides insights in on metastasis development.

## 4. Application of PAFC for the Detection of CTCs

### 4.1. Detection of Melanoma CTCs

Cutaneous melanoma is an almost ideal target for PAFC because this technology can provide routine, label-free, *in vivo* clinical assessment of CTCs for earlier detection of this most aggressive malignancy, which often progress to incurable metastasis at a very early stage. The label-free nature of PAFC applied to melanoma means that PACF can be translated to clinical application much sooner for melanoma than for other cancers, with obvious beneficial public health consequences for this devastating disease. In this study, the overexpression of melanin was used as an intrinsic melanoma cell marker, which provides high PA contrast in that NIR range against the background signal of blood. 

#### 4.1.1. Label-Free Detection

**Figure 10:**
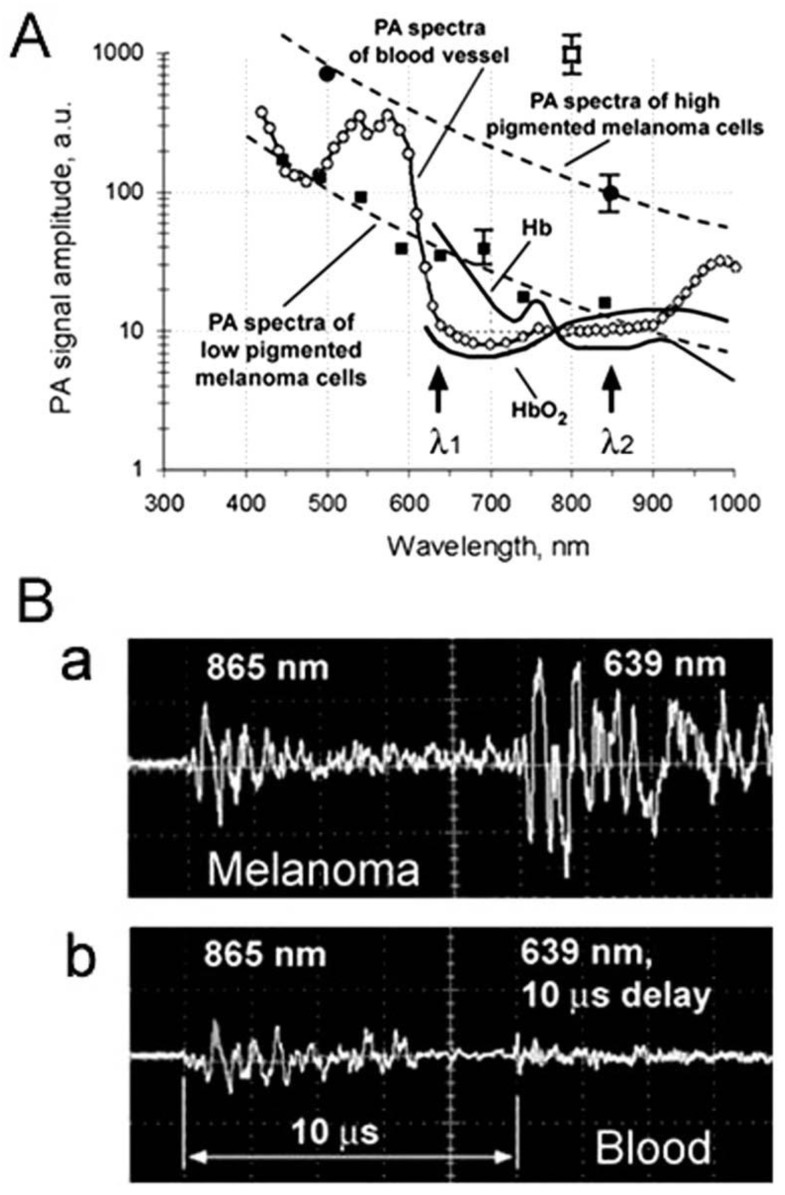
(**A**) PA spectra of ~50 μm diameter veins in mouse ear (*empty circles*); the average standard deviation for each wavelength is 20%. The absorption spectra of the B16F10 cells (*dashed curves*) were normalized to PA signals from single melanoma cells with strong (*black circle*) and weak (*dark square*) pigmentation; open square indicates signal from melanoma cell with GNRs; fragment of solid curves shows absorption for 100% of hemoglobin and oxyhemoglobin; (**B**) Real-time two-color PAFC (λ_1_ = 639 nm; λ_2_ = 865 nm) to identify melanoma CTCs among RBCs.

PAFC’s diagnostic value *in vivo* has been evaluated after intravenous injection of melanoma cells in healthy nude mice or in tumor-bearing nude mice with naturally produced CTCs. By measuring PA spectra from mouse blood vessels and nearby skin, we determined the optimal spectral range (e.g., 660–690 nm, 770–850 nm, and 1,050–1,070 nm) with maximal spectral PA contrast of melanoma CTCs in blood (Figure 10A) [82]. Real-time, two-color spectral identification of PA signals from melanoma CTCs was performed with two laser pulses at wavelengths of 865 nm and 639 nm with a 10 µs delay between the pulses. Because absorption spectra for melanin and venous blood in selected spectral ranges have distinctive features (decreased and increased absorption with increasing wavelength, respectively) (Figure 10A), RBCs generate permanent PA signals with higher amplitudes at 865 nm than at 639 nm (Figure 10B, bottom). In contrast, melanoma CTCs create PA signals with a higher amplitude at 639 nm than at 865 nm (Figure 10B, top). 

Real-time PA counting of metastatic melanoma CTCs (B16F10) was performed in 50 μm-diameter mouse ear vessels, 200 μm-diameter abdominal vessels, and 0.9 mm-diameter aorta (with a focused ultrasound transducer) during tumor progression in the ear and skin of the nude mouse model. The CTC rates in the vessels examined were 0.05, 2.7, and 91 CTCs/min, respectively, underscoring the higher probability of detecting CTCs in larger vessels with higher flow rates, particularly the aorta, through which nearly the entire blood volume of a mouse (~2 mL) circulates within 0.5–1 min as compared to many hours (up to 2 days) in smaller ear vessels [28]. It should be emphasized that the cell flow rate, even in the mouse aorta, is in the range of 10^7^–10^8^ cells/s which is 10^2^–10^3^ times higher than the rate achieved with modern conventional FC *in vitro* [31,32]*.*

**Figure 11:**
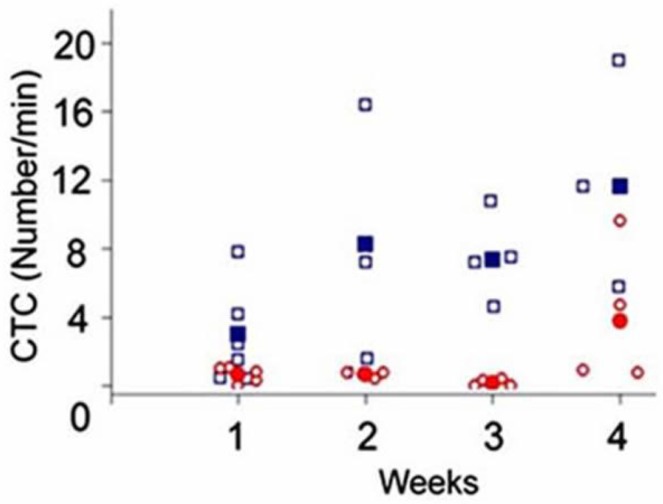
Average melanoma CTC rate in 150 µm-diameter skin vessels as a function of time after B16F10 cell inoculation in the ear (*red open circle*) and skin (*blue open square*); filled red circle and filled blue square, averaged data (905 nm, 30 mJ/cm^2^).

Scanning of a focused laser beam in the vicinity of the ear primary tumor (PA scanning cytometry mode) revealed localized PA signals from migrating individual or clustered melanoma cells in the first week (3–4 days) after tumor inoculation. Metastatic cells appeared in ear microvessels near the tumor in week 2, with no cells detected in the abdominal skin blood vessels. Three weeks later, CTCs appeared in the systemic circulation. This indicated a much greater likelihood of detecting the initial metastatic process in the vicinity of the primary tumor before CTCs disseminated into the larger blood pool. The skin tumor growth rate was faster than that of ear tumors, and CTCs also appeared more quickly in the circulation. In particular, by week 1, 1–4 CTCs/min were detected in the skin vasculature, and as the tumor size increased, the number of CTCs gradually increased (Figure 11) to ~7 CTCs/min and ~12 CTCs/min by weeks 3 and 4, respectively. On one occasion, either PA signals with complex shapes or one large PA signal was observed, which supports the hypothesis of circulating melanoma cells as aggregates. Indeed, optical imaging of ear vessels near a tumor revealed CTC aggregates on the vessel wall, indicating a high probability of CTCs aggregating during intravasation. 

The mice were euthanized, and tissue sections from different organs (e.g., lung, liver, brain, lymph nodes) were examined by immunohistochemical staining (see details in [82]). No evidence of metastasis was found during the first 3 weeks after tumor inoculation for the ear tumor model, while PAFC demonstrated early detection of CTCs at 4 days after tumor inoculation [82,90]. Thus, CTCs can be readily detected with PAFC weeks before conventional techniques show any evidence of detectable metastasis in the tissue samples. 

**Figure 12:**
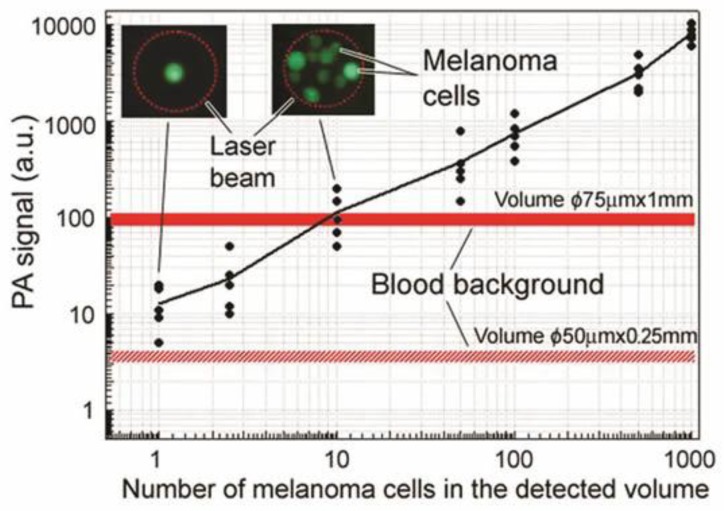
PA signals from human blood samples with different numbers of melanoma cells in the detection volume (850 nm, 150 mJ/cm^2^).

By PA counting rare CTCs in the aorta, we estimated PAFC’s sensitivity threshold at 0.5–1 CTC/mL. This was verified *ex vivo* by assessing the whole blood volume with scanning PT and PA cytometry using unfocused ultrasound transducer (Figure 12) [82]. Specifically, cultured human melanoma cells (HTB-65) were added to anticoagulated blood samples from healthy human donors and subjected to PAFC in microscope chambers containing varying concentrations of melanoma cells (1, 3, 10, 50, 100, 500, and 1,000 cells per unit of volume). The average number of melanoma cells and RBCs were verified by transmission microscopy using thin slides and by fluorescence imaging after staining of melanoma cells with FITC. This study revealed a detection limit at an SNR of 2 of one melanoma cell in the irradiated volume (beam diameter of 50 µm × 0.25 mm [sample thickness]) (Figure 12). For a larger irradiated volume (beam diameter of 75 µm × 1 mm [sample thickness]), the detection limit was a minimum of 10–20 CTCs. The focused ultrasound transducer significantly (20–40 times improved this limit by decreasing blood background levels. 

This unprecedented threshold sensitivity (~1 CTC/mL) in an animal model established PAFC as a powerful research tool for studying behaviors and roles of CTCs in metastasis development at an early cancer stage. PAFC’s sensitivity can be improved 100-fold (*i.e.*, ~1 CTC/100mL) by examining the larger blood volume of humans, which is unachievable with existing assays.

Intravenous injection of RBCs and WBSs labeled with ICG (approved for human use) and melanoma cells in different functional states revealed their different clearance rates [28]: 1–2 min for necrotic cells, 5–15 min for apoptotic cells, 30–60 min for highly metastatic cells (B16F10), 1–2 h for tumor cells with lower metastatic activity (e.g., SK-MEL-1), and 3–5 days and 1–3 days for normal RBCs and WBCs, respectively. By intravenous injection of ICG and trypan blue (broadly used for viability tests *in vitro*), we revealed rare, notable PA signals *in vivo* that were associated with necrotic cells taking up dye directly into the bloodstream [28,91]. Thus, in addition to the detection of apoptotic cells [79], we demonstrated PAFC’s potential for detecting circulating necrotic cells. On the basis of the differences in clearance times, we estimated the percentages of necrotic, apoptotic, and normal CTCs as 5%, 76%, and 19%, respectively. This is important for cancer-related applications, including identification of viable CTCs that are the putative drivers of metastasis, and for monitoring the response to therapies based on activation of apoptotic and necrotic processes in a tumor cells through different mechanisms. 

Acquisition of PA signals from individual melanoma cells *in vitro* and *in vivo* demonstrated PAFC’s capability to detect 60%–70% of strongly pigmented cells (e.g., B16F10) and 20%–40% of weakly pigmented cells (e.g., SK-MEL-1 and C8161) in 50–200 μm-diameter vessels [82,90]. These data obtained by comparing PA signals from non-labeled and labeled NPs in melanoma cells can be used to estimate the false-negative rate, and hence to correct experimental data. These data are in line with the results obtained by integrated PAFFC with B16F10-GFP and C8161-GFP melanoma cells (Figure 9) [49]. This study allowed us to estimate the false-negativity rate for label-free PAFC in the range of 10%–20% and 40%–80% for strongly and weakly pigmented melanoma cells, respectively. Thus, the low intrinsic PA contrast of weakly pigmented melanoma cells impeded the estimation of CTC rates, emphasizing the importance of non-linear PAFC.

From the obtained data, the sensitivity compared with the time-of-observation thresholds can be roughly estimated as the following: in 50 μm-diameter vessels: 1 CTC/1 mL in ~14 h; in 100–300 μm-diameter vessels: 1 CTC/10 mL in ~1 h; and in 1–1.5 mm-diameter vessels: 1 CTC/100 mL in ~20–60 min. Thus, even in ~200 μm-diameter vessels, 1 h of blood monitoring allows us to improve the sensitivity 10-fold lower than the current ~1 CTC/mL seen with existing assays. As mentioned previously large distances between individual CTCs in blood flow, even at relatively high CTC concentrations, (e.g., ~1 cm in 1 mm-diameter blood vessels at 10^2^ CTC/mL) provide less strict requirements for PAFC resolution. 

We believe that the label-free PAFC technology may have tremendous clinical significance due to its high sensitivity and lack of a time-consuming labeling procedure. It can indicate the presence of CTCs in the blood at extremely low concentration, much below the sensitivity threshold of other methods. Intrinsic PA contrast of melanin can be used in melanoma-related research fields including (1) monitoring of melanoma CTC dissemination in blood and lymph flow; (2) study of cell pigmentation and melanin synthesis *in vitro* and *in vivo*; and (3) correlation of CTCs count in the primary tumor, lymphatics, and SLNs. Clinical scenarios may include (1) blood screening for early CTCs before development of metastases, (2) testing for cancer recurrence, (3) individualized assessment of therapeutic efficacy (e.g., chemotherapy, radiation therapy, or immunotherapy) through real-time CTC counts, and 4) potential metastasis prevention by well-timed therapy, including *in vivo* non-invasive laser PT killing of melanoma CTCs. 

We also identified ways for further improving PAFC: (1) decreasing background signals from blood through changes in its oxygenation, osmolarity, and hematocrit, within physiological norms [82]; (2) assessing CTCs in deep, large vessels (e.g., jugular vein) with a focused ultrasound transducer [28]; (3) targeting of melanoma CTCs *in vivo* with MNPs conjugated with specific antibodies followed by magnetic-CTC enrichment [90]; (4) producing melanin in melanoma and even non-melanoma cells (e.g., breast cancer) via transfection with tyrosinase-activating plasmids(e.g., pEGFP-wt-TYR) [63]; and (5) increasing the PA contrast by drug-induced activation of melanin synthesis in melanoma cells [82]. In particular, we demonstrated that a conventional drug, paclitaxel, at typical doses (e.g., 5 µg/mg, 2 day incubation in suspension) led to activation of melanin synthesis in melanoma cells and, in turn, increased PA signals in 10–15-fold, particularly in B16F10 cells [82]. Thus, some drug therapies can increase PAFC sensitivity in the detection of weakly pigmented melanoma. 

#### 4.1.2. Study of CTC Release from the Primary Tumor during a Medical Procedure

**Figure 13:**
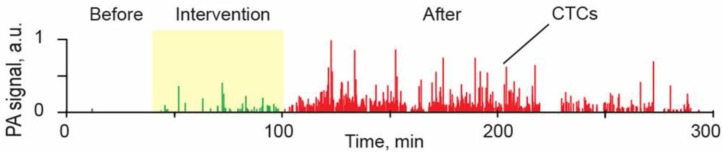
Typical PA signal trace produced by melanoma CTCs (B16F10-GFP) in microvessels of the mouse ear before, during, and after application of 120 g pressure on an ~5 mm-diameter skin tumor [28,29].

For many years oncologists believed that some medical procedures may provoke metastasis; however, no convincing data were reported. Using label-free, real-time PAFC in a melanoma-bearing mouse model, we discovered that palpation, biopsy, and conventional and laser surgery (Figure 1, left) may either initiate release of CTCs into the blood, where they previously were not present, or dramatically increase (10–30-fold) CTC counts above the previous level; both of which can increase the risk of metastasis [29]. In particular, the pressure of a 120 g weight notably increased the CTC count (Figure 13), which eventually led to the appearance of lung metastases at week 3 after tumor implantation, as compared to no metastasis without the pressure of palpitation. Blood vessel damage in a tumor during a biopsy was modeled by a small, scalpel incision or laser tumor treatment, and led to the appearance of CTCs either immediately or after a few hours. In contrast, complete melanoma tumor resection with a positive margin led to the quick (within a few hours) disappearance of previously observed CTCs. However, in several cases, CTCs appeared again in the circulation a few weeks after surgery, which might indicate possible CTC release from metastasis in distant organs. Although an animal model was used in this study, our results may warn oncologists to take some precaution during physical examination, to create a careful surgical plan, and to stress the importance of adjuvant or preventive anti-CTC therapy during primary tumor treatment. Patients should avoid wearing tight clothes to avoid skin pressure above the tumor. *In vivo* PA detection of the appearance of intervention-induced CTCs in the circulation could be used for early diagnosis of small tumors undetectable with conventional methods [29]. 

#### 4.1.3. PA-PT Theranostics of Melanoma CTCs with Nonlinear PAFC

**Figure 14:**
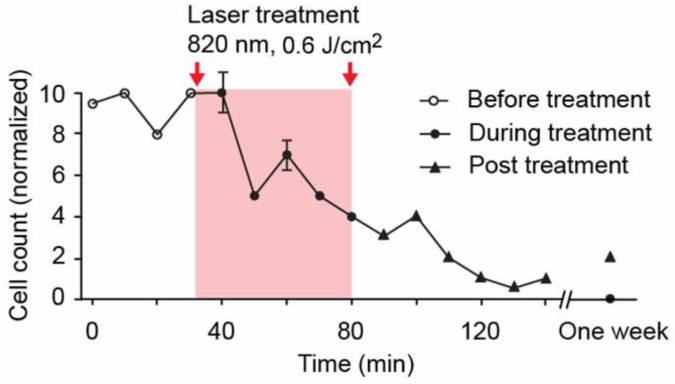
Theranostic of melanoma CTCs integrating PA detection, PT killing, and PA assessment of therapeutic efficacy through a decrease in the CTC count [28].

Our data show that PAFC can be used both as a research tool in animal models to provide insights on the potential of various therapies to elicit CTCs and thus increase the risk of metastasis and as a clinical instrument for personalized cancer diagnosis and for guidance of appropriate therapy. In particular, when we exposed an mouse abdominal vessel to an 820 nm laser, increasing the energy fluence from 60 mJ/cm^2^ to 600 J/cm^2^ led to an ~6-fold increase in PA contrast of the CTCs above the RBC background [82,95,102]. This phenomenon was associated with the generation of laser-induced nanobubbles around overheated, strongly absorbing melanin nanoclusters in melanoma cells, which served as non-linear PA signal amplifiers, as compared to linear PA signals from RBCs with a homogenous hemoglobin distribution (*i.e*., with no nanobubble formation). On the other hand, CTC rates gradually decreased from 12 CTCs/min to 1–2 CTCs/min over a 1 h monitoring period (Figure 14). This effect was also associated with the generation of nanobubbles as melanoma cell killers [82,102]. Nevertheless, the CTC rate later gradually increased almost to levels initially detected, indicating the appearance of new CTCs from the primary tumor or metastasis. These data demonstrate the great potential of PAFC and PT techniques [71] for guiding cancer therapy and blood purging *in vivo* by periodically exposing blood vessels in different locations to laser [28,94,95]. Further study could determine whether this new treatment is effective enough to be used alone or whether it should be used in combination with chemo- or radiation therapy.

#### 4.1.4. Magnetic Capture of Melanoma CTCs *in Vivo*

The combination of conventional FC with separation, isolation and enrichment methods revolutionized the diagnosis and therapy of diseases. Various physical cell properties, including size, motility, and electrical dipole moments, as well as optical and magnetic qualities, have been exploited for this purpose. In particular, specific cells (e.g., CTCs, bacteria) or biomolecules (e.g., proteins, DNA) in biological fluids such as blood, urine, and cerebrospinal fluid, were labeled with magnetic microbeads and MNPs. They were then separated and enriched from the sample flow by a magnetic field (see [81] and references there]. To date, these techniques have been used only in *ex vivo*, while *in vivo* applications are limited to static objects only. We demonstrated, for the first time, the application of magnetic capture and enrichment of CTCs directly in the bloodstream [81,85,90,94].

**Figure 15:**
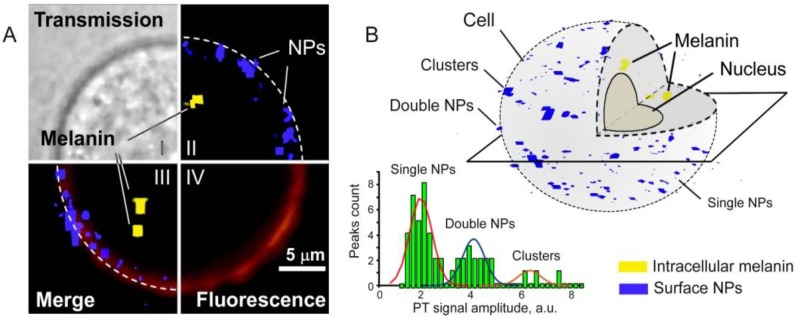
(**A**) Transmission (I), confocal PT at 532 nm (II), fluorescence (IV), and combined (III) images of SK-MEL-1 cells labeled with anti-MCSP MNPs and anti-MCSP-PE (dye); (**B**) 3-D distribution of surface MCSP receptors (blue) and intracellular melanin (yellow). These data were obtained by D. Nedosekin.

Specifically, as PAFC could miss weakly pigmented melanoma cells, we further evaluated its capability to detect melanoma CTCs targeted *in vivo* in the circulation by functionalized 50-nm MNPs. We selected the human melanoma cells (SK-MEL-1) expressing MCSP receptors [2]. MNPs were conjugated with monoclonal anti-human MCSP Abs. MSCP expression of the melanoma cells was assessed by conventional FC and fluorescence imaging of cells labeled with phycoerythrin (PE)-conjugated monoclonal antihuman MCSP-Abs. To verify target specificity and efficiency *in vitro*, we applied also our new scanning two-beam (pump-probe) confocal PT microscopy [63] integrated with a fluorescence module for simultaneous 3D imaging of the distribution of intrinsic intracellular melanin and surface MCSP receptors. Spatial coincidence of fluorescence and PT signals indicated the ability of functionalized MNPs to specifically label surface MCSP receptors (Figure 15A, III). Specifically, 3D PT imaging allowed us to distinguish low level expression of melanin located in the cytoplasm from MCSP receptors on the cell surface (Figure 15A, II). By peak signal analysis, we distinguished single NPs from clustered NPs around single and clustered receptors, demonstrating marker spatial heterogeneities. In weakly pigmented cells without NPs, the PT technique detected 16% of all melanoma cells due to relatively low melanin expression. After labeling of cells with MNP-MCSP conjugates, PT signals were detected in 68% of melanoma cells, suggesting an advantage of combining molecular labeling and intrinsic markers. These results were in an agreement with conventional FC, which showed MCSP expression in 51% of cells (*vs.* 52% obtained with PT microscopy) compared to 3%–5% with non-specific binding.

**Figure 16:**
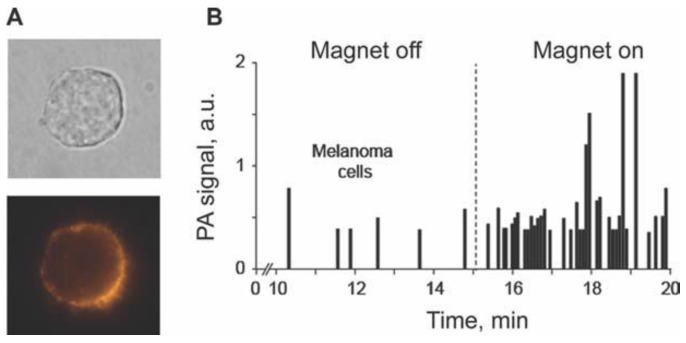
*In vivo* targeting of circulating SK-MEL-1 human melanoma cells in mouse blood. (**A**) MSCP expression by SK-MEL-1 cells, transmission (top) and fluorescence (bottom) images of a cell labeled with anti–human MSCP-Abs conjugated with PE fluorescent dye; (**B**) PAFC trace in tumor-bearing mouse model 10 min after CTC targeting by 50 nm MNPs conjugated with anti-human MSCP-Abs before and during magnet action. Laser wavelength, 1,064 nm; fluence, 100 mJ/cm^2^; pulse repetition rate, 10 kHz [90].

The half-life of MNPs circulating in mouse blood after intravenous injection of a high concentration of MNPs (10^11^ NPs/mL) was found to be 20 ± 5 min. No PA signals were observed after intravenous injection of an optimized, relatively lower (100-fold) concentration of MNPs, suggesting that signals from unbound or non-specifically bound MNPs were weaker than background signals from blood. After injection of 10^5^ unlabeled cancer cells followed by injection of MNPs, no PA signals were immediately observed. However, a gradual increase in the rate of high PA signals was observed within next 20–40 min after injection of the MNPs. These data suggest that targeting of CTCs by MNPs increased their localized concentration and provided PA contrast sufficient for detection of a single CTC. Gently attaching a 0.7-Tesla magnet to the mouse ear at a distance of 0.3 mm from the laser spot led to an immediate increase in both the amplitude and the rate of PA signals (Figure 16B). It is likely that labeled cells were completely trapped or slowed down in the strong magnetic field gradient. As a result, under the action of the magnet, the rate of appearance of intensive PA signals associated with melanoma cells circulating *in vivo* increased from 1.1 cells/min to 12 cell/min. 

### 4.2. Detection of Breast CTCs in Blood

#### 4.2.1. Multiplex Molecular Targeting, Magnetic Capture, and Two-Color Detection of Bulk Breast CTCs

**Figure 17:**
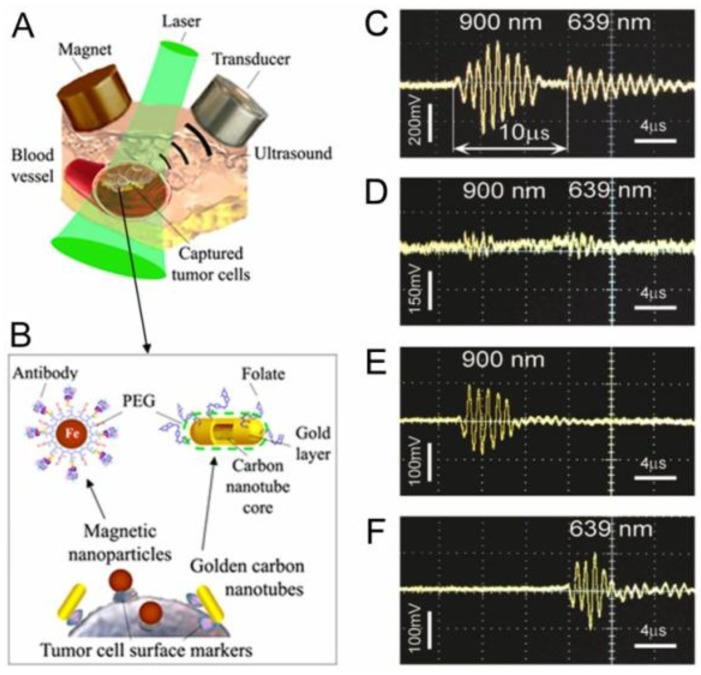
*In vivo* magnetic capture and two-color PA detection of breast CTCs. (**A**) Schematic showing the detection method. The laser beam is delivered either close to the external magnet or through a hole in the magnet with a fiber-based delivery system. (**B**) Schematic of targeting breast CTCs with MNPs (a 10 nm core, a 2 nm layer of amphiphilic triblock copolymers, and the ATF of the uPA and GNTs (12 nm × 98 nm) coated with PEG and folic acid. (**C**–**F**) Typical PA signals at different wavelengths from CTCs labeled with MNPs and GNTs (**C**), GNTs only (**D**), and MNPs only (**E**). (**F**) PA signals from blood vessels only [81].

We integrated two-color PA detection and magnetic enrichment of breast CTCs in circulation, using advanced GNTs [80] and MNPs conjugated with breast cancer markers (Figure 17A,B). In light of the limited expression of most cancer markers, we applied a multiplex targeting strategy for the detection of CTCs. Specifically, we used duplex molecular targeting of MDA-MB-231 human breast cancer cells, which are positive for urokinase protease-activated receptors (uPAR) and folate receptors (FRs). It has been demonstrated that 60%–90% of breast cancers express uPAR (~10^5^ receptors/cell *vs.* 2,500 receptors/cell for normal human epithelial cells) and 80%–90% express FRs [81]. GNTs having an absorption maximum at 900 nm and a minimum at 639 nm were coated with polyethylene glycol (PEG) and conjugated with folates. As the second NP, 10-nm MNPs coated with PEG and amphiphilic triblock polymers were conjugated with the amino-terminal fragment (ATF) of a urokinase plasminogen activator (uPA), which is a high-affinity, natural ligand for uPAR. MNPs have absorption in the broad NIR range; nevertheless, their absorption spectrum is different from that of GNTs. PA and PT scanning cytometry, in combination with fluorescence imaging, revealed that this NP cocktail exhibited the best targeting efficiency (96%) *in vitro* in a blood sample spiked with rare tumor cells and a negligible level (~6%) of background PA signals from unbound or non-specifically bound NPs.

**Figure 18:**
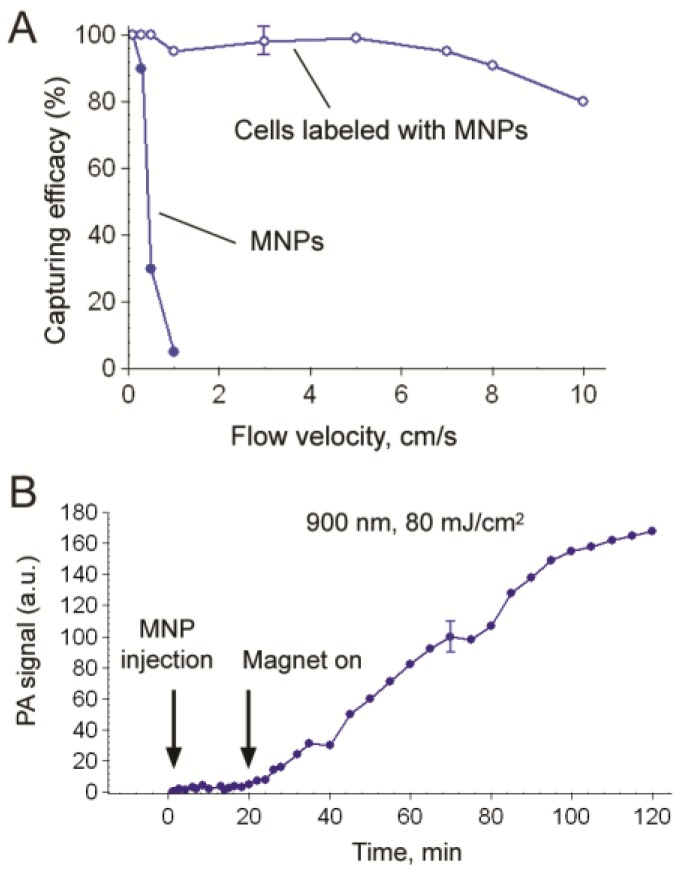
(**A**) Capturing efficiency for MNP-labeled cancer cells and free MNPs at different flow velocity of PBS in tube with diameter of 0.5 mm; (**B**) PA signals from CTCs in 200–300 µm abdominal mouse skin vessels obtained with fiber schematics at week one tumor development before and after magnet action [81].

Attaching a magnet with a field strength of 0.39 Tesla to the testing tube with diameter of 0.12, 0.5 and 0.8 mm captured 10 nm MNP-labeled cells in phosphate buffer saline (PBS) (Figure 18A,B) at a broad range of flow velocity (0.1–10 cm/s) and this was accompanied by strong PA signals from the area under magnet compared to the signals outside magnet with rare uncaptured cells and unbound MNPs [82]. Introduction of free MNPs in high concentration (10^11^ MNPs/mL) with a small number (5–10) of MNP-labeled cells demonstrated that both MNPs and MNP-labeled cells were captured at flow velocity of 0.1 cm/s. Increasing the velocity to 3 cm/s removed most of the free MNPs but the MNP-labeled cell remained captured. Because magnetic force is proportional to particle number, the randomly distributed free MNPs were more effectively removed by flow drag forces than the labeled cells that contained a higher local MNP concentration or dense MNP clusters. As a result, labeled cells were captured effectively up to flow velocity of 5–10 cm/s while free MNPs were captured only at low velocity of 0.3–0.5 cm/sec (Figure 18A).

In animal-model experiments, we demonstrated the potential to capture cells with MNPs in flow at velocities up to 5–10 cm in up to 1–2 mm vessels, using a magnet with field strength of 0.39 Tesla. We also discovered that a fiber-based PA-magnet probe provided the opportunity for PA- (or PT-) guided magnetic manipulation of MNPs in cells [85]. In particular, PT and PA signals from individual cancer cells after magnet action increased in amplitude up to 5–10 times. This magnet-induced signal amplification was associated with enhanced clustering of MNPs under magnet exposure as a PT and PA signal amplifier. These phenomena were verified by fluorescence imaging of MNPs with fluorescent labels, which demonstrated a significant increase in localized fluorescence intensity within single cells from endocytosed MNPs, as compared to the relatively homogenous spatial fluorescent light distribution before the magnetic action. One possible mechanism responsible for the appearance of localized intracellular zones with clustered MNPs is associated with extensive localized concentration of MNPs under a magnet near cellular structures (e.g., membrane and organelles) acting as the mechanical obstacles. Magnet-induced NP clustering can be accompanied by a spectral shift in PT and PA spectra that can be used to spectrally identify MNPs and CTCs labeled with MNPs. 

**Figure 19:**
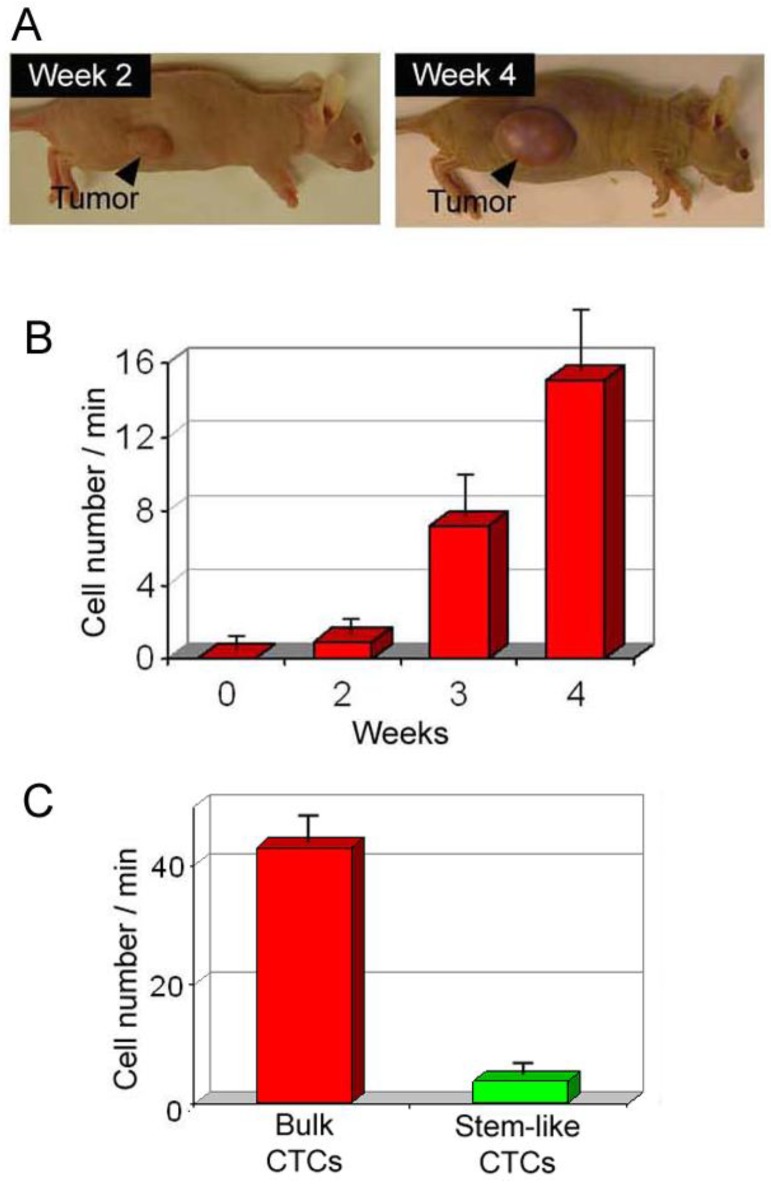
PA detection of bulk and stem-like breast CTCs in tumor-bearing mice. (**A**) The size of the primary breast cancer xenografts at different stages of tumor development; (**B**) Average rate of bulk CTCs in mouse ear vein over a period of several weeks. (**C**) Average rate of CTCs associated with bulk folate^+^/uPAR^+^ CTCs and folate^–^CD44^+^ stem-like CTCs in200 µm-diamater abdominal skin blood vessels in a mouse model of breast cancer (at week 4).

At weeks 2, 3, and 4 of tumor development in the mouse models (Figure 19A), a cocktail of conjugated NPs was injected intravenously into the circulation. Transient PA signals simultaneously at both 639 nm and 900 nm indicated targeting of both cancer markers (Figure 17D). Additionally, we observed rare (a low percentage of the total number of signals) transient PA signals at only 900 nm or 639 nm (Figure 17E,F), which may be associated with targeting the small number of CTCs that expressed one of the selected markers. Blood produced weak background signals with comparable amplitudes at both wavelengths (Figure 17D). We did not detect any PA signals with consistent amplitudes that were above the background signal of blood, indicating a negligible background from unbound NPs. PA monitoring of targeted CTCs at 20 min after injection (to allow clearance of most unbound NPs) showed that the CTC rate in ear mouse as compared with abdominal vessels (CTCs/min) increased from (0.9 + 0.3)/(6 + 2.1) at week 2 to (7.2 + 0.3)/(26 + 2.1) at week 3 and to (15.1 + 2.7)/(47 + 6.4) at week 4 (Figure 19B). These data approximately correlated to the stage of primary tumor progression. Attaching a magnet with a 0.39-Tesla field strength at week 1 led to immediate increases in both PA signal amplitude and rate and changed the character of the PA signal from infrequent flashes to a continuous increase of permanent PA signals up to 88-fold within 1.5 h, indicating successful magnetic capture of CTCs (Figure 18B) [81]. In the clinical application of this method, patients may carry a magnet attached to selected peripheral vessels (e.g., in the wrist area) to trap CTCs, with quick PA detection of the trapped CTCs, and, if necessary, localized PT treatment or removal of CTCs by using a syringe-based system for further molecular analysis. 

The PT and PA contrast of MNPs in the NIR range is significantly (≥10-fold) lower than that of gold-based NPs, such as GNTs and GNRs. Nevertheless high senility PAFC provides detection with intrinsic absorption [81]. Taking into account linear PA signal enhancement using gold NPs with a dense layer of polymer [76] and silica [111], we demonstrated that at a low laser energy level silica-MNPs (siMNPs) [94] provided 8- to 2.5-fold greater linear PA-PT signal amplification than MNPs alone. At a higher laser energy level, we observed a giant (20- to 35-fold) non-linear PA-PT signal amplification. This can be explained by the favorable thermal acoustic- and nanobubble-related properties of siMNPs for the generation of non-linear PT and PA effects [94]. 

#### 4.2.2. Flow Cytometry Platform for Detection of Circulating Cancer Stem Cells in Blood

It is believed that a small population of cancer cells (3%–5%), called tumor-initiating or cancer stem cells, may be a cornerstone of metastatic initiation and progression due to their extensive self-renewal capacity, tumorigenicity, and multipotentiality associated with drug and radiation resistance [1,2,3,4,5,6,7,8,9]. Thus, these cells could be a novel and crucial target for diagnosis and therapy. However, little is known about subpopulations of these cells that can enter the peripheral circulation and migrate to distant sites, forming metastases. Because an extremely low concentration of stem CTCs is expected in the circulation, we proposed using high-sensitivity PAFC to detect stem CTCs [85]. Specifically, *in vivo* targeting of breast CTCs with a stem-like phenotype, which are naturally shed from the parent tumor in mouse models, were performed with functionalized GNTs and MNPs. Data *in vivo* were verified *in vitro* with scanning PA and PT cytometry. We demonstrated proof-of-concept *in vitro* that PA diagnosis can be integrated with targeted PT eradication of individual stem CTCs. 

In a preliminary study *in vivo*, GNTs and MNPs conjugated with folic acid and antibodies to CD44 were selected for the detection of stem-like CTCs. To identify bulk CTCs, we used the markers described in Section above 4.2.1. At week 4 after tumor inoculation, when metastatic disease was well established in the distant organs (e.g., liver), MNP-folate and GNT-CD44 conjugates were separately injected into a mouse tail vein. To allow time for effective labeling of CTCs in the bloodstream and washing out of unbound NPs, PA monitoring of blood vessels began 30 min after injection. As expected, readable flashing PA signals above the PA background of blood were detected within 2 h of the observation. This indicated that stem-like CTCs represented ~10%–15% of the bulk CTCs (Figure 19C). To the best of our knowledge, we demonstrated, for the first time, that a multifunctional PA-PT nanotechnology-based-platform has the potential for ultrasensitive PA molecular detection of stem CTCs *in vivo*. The interpretation of the obtained data at the current stage requires further study, which is now in progress in our laboratory. It includes exploring the role of the folate receptor and adding CD24 and CD45 markers to increase detection and specificity of stem-like CTCs and to exclude possible false-positive signals from leukocytes, respectively.

In a preliminary study, we explored molecular targeting of melanoma-initiating stem-like cells using ABCB5 markers previously shown to be associated with metastatic disease progression in melanoma patients [20,24]. ABCB5^+^ cells were targeted by GNRs (maximum absorption at 671 nm) conjugated with antibodies against ABCB5 markers, while bulk CTCs with GFP were detected by FFC. By comparing PAFC and FFC data, we estimated the percentage of ABCB5^+^ cells in circulation (up to 20%–40%) as compared to primary tumor cells (~5%), which suggests a more effective invasion rate of tumor-initiating cells in circulation.

### 4.3. *In Vivo* Lymph Flow Cytometry

#### 4.3.1. PA Detection of Tumor Cells in Lymph Flow and Sentinel Lymph Nodes

Lymph is the most common medium in the human body, but it is currently poorly understood. Numerous studies have demonstrated the high importance of cell trafficking by lymph flow for the functioning of the whole organism. In particular, cancer cells from the primary tumor in many cases can disseminate through lymphatics at least at the initial stage. However, compared with blood vessels, lymph vessels are tiny structures with colorless fluid moving at low pressure and containing a relatively low concentrations of cells. Therefore, finding lymph channels requires mapping with contrast agents (Figure 3A–C), and lymph sampling yields only a few microliters at a time or requires long-term cannulation using special skills. All of these factors make the detection of CTCs in lymph (also called disseminated tumor cells [DTCs]) by conventional *in vitro* methods extremely difficult, and these procedures are performed only in a few laboratories and clinics. 

**Figure 20:**
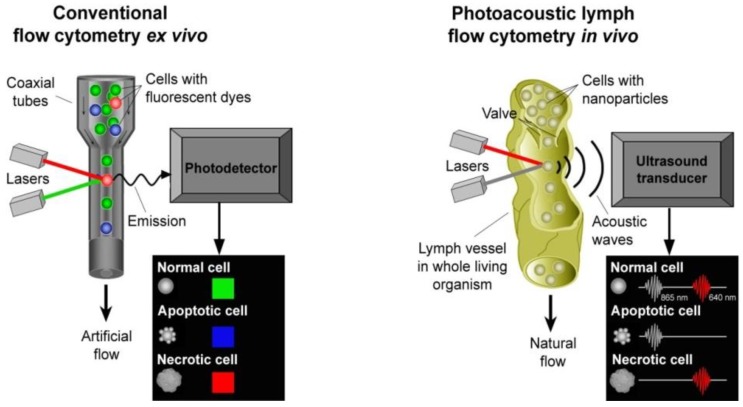
Comparison of conventional flow cytometry *in vitro* (left) and *in vivo* PA lymph flow cytometry (right) for detection of cells in different functional states.

**Figure 21:**
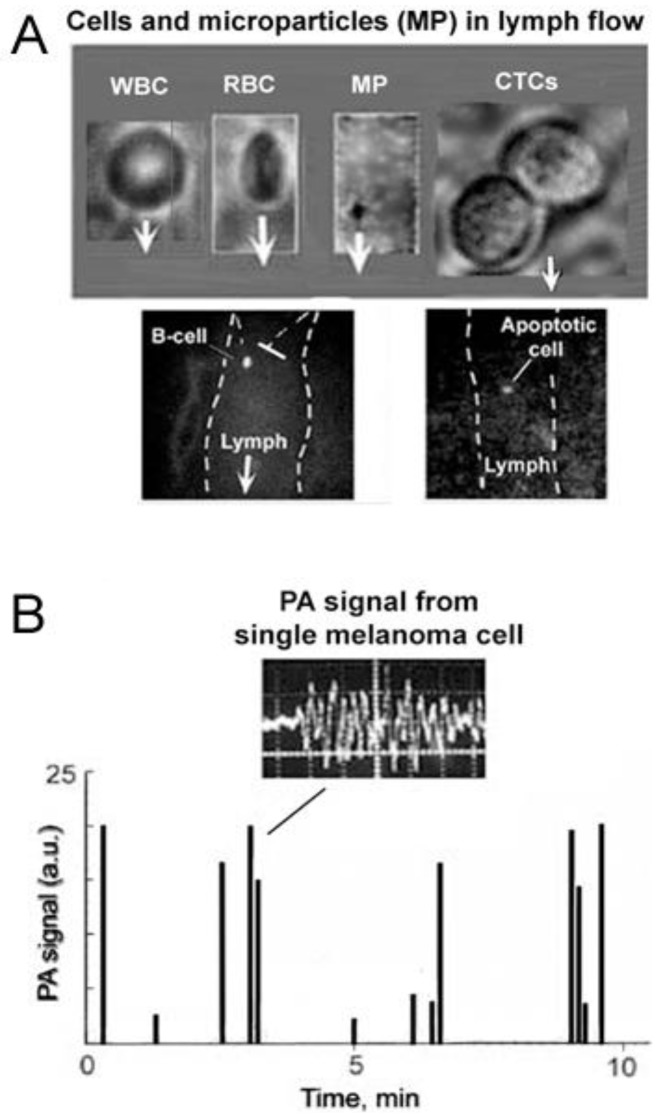
*In vivo* lymph flow cytometry. (**A**) *In vivo* FC image with transmission and fluorescence techniques for identification of a WBC, an RBC, a microparticle (MP; likely chylomicron), CTCs, B lymphocytes, and apoptotic WBCs in lymph flow (magnification, 40×; image rate, 500–2,500 fps). (**B**) Application of label-free PAFC for detection of melanoma CTCs in an ear lymphatic in a preclinical animal model of metastatic melanoma (ear tumor in nude mice; week 2 after inoculation); insert: non-compressed time-resolved PA signal.

The advantage of *in vivo* PAFC of lymph (Figure 20, right) is the ability for label-free detection of cells with intrinsic PA contrast agents, such as melanoma CTCs. In PAFC, the colorlessness of lymph, commonly accepted as a limitation for diagnostic techniques, is transformed into a diagnostic advantage, providing a low PA background. Using integrated PA-fluorescence techniques, we have demonstrated detection and imaging of RBCs and WBCs (e.g., B cells) in normal and apoptotic states (Figure 21A), as well as pigmented melanoma cells in pre-nodal lymphatics (Figure 21B) and SLNs [28,79,83]. In the mouse ear melanoma tumor model, colorless ear lymph vessels were visualized by injecting Evans-blue (EB) contrast dye into peritumoral tissue areas (Figure 3C). The absorption of EB is within the visible spectral range (below 680 nm) and does not interfere with the NIR region (820–900 nm) used for label-free detection of pigmented melanoma cells. Metastatic cells were observed in ear lymph vessels during a pre-metastatic stage of tumor development (day 7 after inoculation; 0.26 ± 0.05 PA signals/min). Further increases in the detection rate of metastatic cells over the course of the next 2 weeks to 2.13 ± 0.3 cells/min were associated with the development of metastasis in SLNs. At the WBC rate of 10^2^ cells/s in 200 μm-diameter vessels, PAFC was able to detect a single melanoma cell during 3 h of observation; this rate is approximately equivalent to one melanoma cell against the background of 10^6^ WBCs. This unprecedented sensitivity threshold in lymphatics *in vivo* is unachievable with existing techniques. 

#### 4.3.2. *In Vivo* PT Imaging of Cells in Lymph Flow

**Figure 22:**
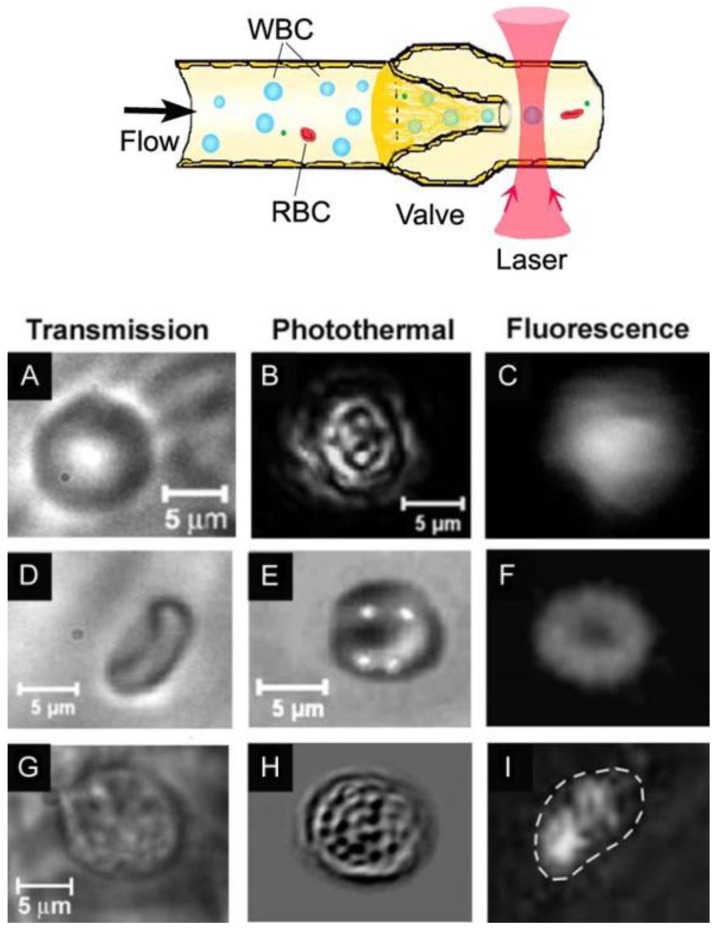
(Top) Schematic for the natural focusing of cells in lymph flow. (Bottom) transmission digital microscopy (TDM) (**A,D,G**), PT (**B,E,H**), and fluorescence (**C,F,I**) images of (top row) leukocytes, (middle row) RBCs, and (bottom row) K562 cancer cells in lymph flow *in vivo*.

PAFC was first combined with PT flow cytometry (PTFC) [69,73]. PTFC alone also demonstrated the capability to image blood cells and CTCs in lymph flow. To obtain high-resolution images with minimum distortion due to spatial fluctuations of cells in lymph flow (up to 50 µm in 150 µm-diameter lymph vessels), cells were imaged immediately after passing through a lymphatic valve (Figure 22, top), which played the role of a natural nozzle focusing the cells near the vessel axis [79]. Thus, lymphatic valves provided a natural type of hydrodynamic focusing (a term used in FC *in vitro* involving an artificial nozzle) to limit lateral fluctuation of cells to within a few micrometers. With such focusing and synchronization of high-speed imaging with vessel and valve function, we obtained high-resolution (300–500 nm) images of cells, including lymphocytes, RBCs, and cancer cells (leukemia, K562) (Figure 22, bottom), as they flowed at relatively high speeds (up to 4 mm/s), especially during the valve-closure stage of vessel constriction. PT imaging revealed specific absorbing cellular structures (Figure 22B,E,F) associated with the spatial distributions of cytochromes in lymphocytes and cancer cells and of hemoglobin in RBCs that were not visible with transmission microscopy (Figure 22A,D,G). Fluorescence images showed high image contrast (Figure 22C,F,I) similar to that of PT images, although leukocytes required labeling with rhodamine 6G, RBCs with FITC, and K562 cells with MitoTracker (Life Technologies Corp., Grand Island, NY, USA). Unlike TDM, PT imaging showed no background noise around the cells due to scattering effects. 

Label-free PTFC allowed us to obtain the images of cancer cells moving at velocities of 0.5–1.5 mm/s (Figure 22G) and distinguish them from RBCs and WBCs with distinctive shapes and PT-image structures associated with hemoglobin and cytochrome distribution in RBCs and WBCs/cancer cells, respectively. In addition, RBCs and WBCs can be identified and removed from analysis through the difference in their PT cooling times (6–10 µs for 4–5 µm RBCs and 20–25 µs for 7–8 µm lymphocytes, with amplitudes 20–30 times higher in RBCs) [69]. We also discovered an opportunity to combine PTFC with TDM and fluorescence imaging (WBCs and RBCs were first labeled by rhodamine 6G and FITC, respectively) to provide cell shape, although the local image structures were hardly visible. 

Fluorescence imaging speed is currently limited to <1,000 fps, which is less than optimal. Therefore, we have also made a concerted effort to develop PT imaging (pump beam, 532 nm, 10–30 kHz; and probe beam, 671 nm, 20–60 kHz) with an unprecedented high speed of up to 10^4^ fps (10^2^-fold greater than existing PT-PA techniques) for single cells of interest (e.g., RBCs) at a flow velocity of 2 m/s without notable motion distortion of the image during a 10-ns laser pulse. We believe that PT techniques will open new opportunities in lymphatic research including (1) quantitative monitoring of normal (e.g., WBCs, macrophages, dendritic cells) and abnormal (e.g., metastatic) cells in afferent lymph flow; (2) label-free estimation of a cell’s functional state (e.g., apoptosis); and (3) the cellular response to different interventions, such as pharmaceuticals, nicotine, lasers, or γ-radiation. In addition, PT diagnostics can be integrated with PT killing of CTCs (theranostics).

### 4.4. Detection of Cancer Cells in the Blood Stream, Lymph Flow and Sentinel Lymph Nodes; Theranostics of Lymphatics

It has been known for some time that lymph and blood vessels are common pathways for the dissemination of cancer cells from the primary tumor and that tumor cells may pass from one system to another through numerous anatomical interconnections between lymph vessels, SLNs, and the blood circulation (see [79] and references there). Nevertheless, until now, CTCs in the lymph and blood systems have been studied separately. Moreover, most efforts have focused on examination of CTCs in the blood system, while studies of the metastatic process in lymphatics was paid much less attention. 

Recently, we integrated *in vivo* blood and lymph PAFC, PA lymphography (study of lymph flow), and PA scanning cytometry (study of non-moving cells), and demonstrated a potential of this platform for real-time *in vivo* quantitative monitoring of CTCs in blood, DTCs in pre-nodal lymphatics, and SLNs with the use of intrinsic melanin or functionalized NPs as PA contrast agents in melanoma-bearing and breast tumor-bearing mouse models, respectively [28,83]. This integration of techniques allowed us to define cross-correlations between lymph DTCs, blood CTCs, the size of the primary tumor, and nodal and distant metastases (Table 1) [28]. Specifically, in the pre-clinical mouse melanoma model, we revealed that early metastatic cells in latent metastatic disease (4–7 days after tumor inoculation) are equally disseminated through blood and lymph pathways. However, in a few cases, metastatic cells appeared in lymph vessels at week 1 without any cells being detected in the blood vessels and *vice versa*, suggesting an individualized character of tumor cell dissemination. During week 2, 3.5-fold primary tumor growth was accompanied by a 10-fold increase in the DTC count in lymph flow and by a 6.5-fold increase in the number of PA signals in SLNs as a sign of metastasis development, while many fewer CTCs were observed in blood flow. The association between DTC count and SLN metastasis progression supports lymphatic DTCs as a novel prognostic marker of metastasis.

**Table 1:** Correlation between primary tumor size, metastasis in SLN and number of tumor cells in lymph and blood flow [28].

Time tumor growth	Tumor size, mm^2^	Rate of lymph CTCs, cell/min	Rate of blood CTCs, cell/min	Number of PA signals associated with metastasis in SLNs	Histology
1 week	1.0 ± 0.2	0.26 ± 0.05	0.85 ± 0.03	493	NO
2 weeks	3.6 ± 0.5	2.13 ± 0.30	1.07 ± 0.05	3,188	YES

In addition, we demonstrated PAFC-guided PT purging of melanoma and breast cancer micrometastases in SLNs, which were mimicked by direct injecting cancer cells into SLNs. Targeted detection of breast cancer metastases in SLNs in the necks of the mouse models was performed by injection of GNT-folate conjugates into the mouse ear. At 5 min after the injection, strong PA signals above the background with a contrast (as a ratio of PA signal amplitudes from cells/NPs and background) appeared in the SLN as the NPs were transported through lymph vessels to the SLN to target micrometastases. Subsequent application of therapeutic laser pulses with enhanced energy fluence led to a decrease of these initially PA strong signals to the background level, which indicated laser-induced destruction of the tumor cells. In contrast, when the experiment was repeated with unconjugated GNTs, low PA signals with a contrast of ~3 above the background were observed, suggesting random distribution of GNTs in the SLN volume. To verify these data, *ex vivo* experiments mimicking the lymph node micrometastasis were carried out with GNT-folate conjugates and fluorescent dye for visualization of tumor cells [80]. These results supported the feasibility of the theranostic PAFC-PT platform for *in vivo* detection and killing of metastatic cells in SLNs taking into account. NP clustering in micrometastasis accompanied by red-shift effects and PA signal amplitude enhancement as indicators of molecular targeting. This platform, in combination with microarrays, might enable assessing the biological properties of lymphatic DTCs (e.g., molecular profiles, viability) compared to those of the primary tumor, regional and distant metastases, and blood CTCs during metastasis progression, with a focus on identifying the tumor-initiating cells among the bulk lymphatic DTCs. Expanded knowledge of lymphatic-related metastasis may catalyze a paradigm shift in diagnostic clinical oncology from conventional assessment of early metastasis in SLNs toward lymphatic DTC testing. Taking into account the safe nature of the proposed *in vivo* lymph cancer tests as supplementary (or in some cases as alternative) to conventional blood tests, we anticipate a quick translation of this technology for use in humans.

### 4.5. *In Vivo* Integrated Cerebrospinal Blood-Lymph Flow Cytometry

The dissemination of cancer (e.g., leukemia, lymphoma, breast cancer, and melanoma) into the central nervous system through hematogenous spread, direct release from the tumor itself, or migration along perineural or perivascular spaces is a serious medical problem leading to neurological symptoms (e.g., neoplastic meningitis) and rapid mortality. In particular, the presence of tumor cells in the cerebrospinal fluid (CSF) may serve as a marker of disease progression and early-stage brain metastasis in breast cancer. However, in analogy to lymph, examination of CSF is extremely difficult by conventional techniques. CSF is a colorless body fluid with a total volume of 135–150 mL in adults and circulates through the ventricular system around and inside the brain and the spinal cord. CSF flow has pulsatile “forward-backward” characteristics that correlate with cardiac cycles. Forward velocity in the spinal canal ranges from 10 mm/s in the craniocervical junction to 1 mm/s in the lumbar part of the canal; the velocity of backward flow is approximately two times slower than that of forward flow. CSF function is to bathe the central nervous system and to bridge the vascular and lymphatic systems. The current tools available for detecting CTC spread into the CSF such as cytology, *in vitro* FC, and others (see [97] and references there) are limited by small sample volume and insufficient sensitivity, leading to delays in treatment. 

**Figure 23:**
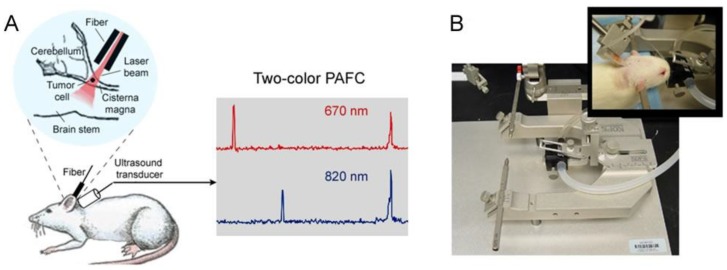
Integrated technical platform for testing CSF *in vivo* and *ex vivo*. (**A**) Schematic of multicolor PAFC with the example of real-time two-color PAFC tracings from CSF; (**B**) The heating stereotaxic table for *in vivo* PAFC and CSF sampling.

Thus, CTCs may spread within the body by lymphatic and/or blood (as discussed above) and/or cerebrospinal pathways that closely interplay with each other. Thus, to understand disease progression, three pathways should be examined simultaneously. However, simultaneous detection of CTCs in these fluids is challenging, and overcoming this challenge could provide knowledge of the mechanisms of metastasis development. We hypothesize that some of these problems could be resolved by increasing the sensitivity and specificity of CSF examination through *in vivo* analysis of a relatively large volume of circulating CSF with non-invasive two-color PAFC using functionalized NPs as PA contrast agents (Figure 23A) [97]. This approach has not been attempted previously. Specifically, we tested the capability of *in vivo* FC to assess the three systems—blood, lymph, and CSF—using an orthotopic xenograft mouse model of human metastatic breast cancer. Because metastasis in the central nervous system is a late event in breast cancer development, PAFC of CSF was performed at week 10 after tumor inoculation. At this point of tumor development, the rate of blood CTCs was estimated by FFC using the CTC-GFP model as 8–12 CTCs/20 min (Figure 23A). Then we found the presence of labeled cells in the SLNs (Figure 23B), indicating 1) the primary tumor’s ability to shed cells into the circulation and 2) the participation of the lymphatic system in dissemination of late breast CTCs. The CTCs in CSF were detected by two-color PAFC at 670 nm and 820 nm on day 1 after injection of the GNR cocktail into the primary tumor. To detect CTCs in CSF, we used a double-targeting strategy with GNR_670_-folate and GNR_670_-EpCam. To increase PAFC’s specificity and reduce the probability for false-positive signals, we added GNR_820_ bioconjugated with ABs to CD45. This receptor was used as a common marker of WBC to distinguish CTCs (CD45^–^) from WBCs (CD45^+^). 

**Figure 24:**
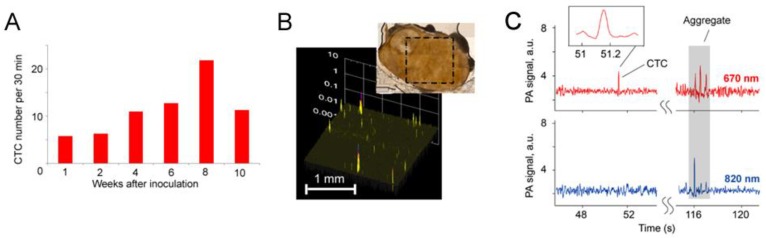
*In vivo* multifluid PAFC of CTCs in blood, lymph, and CSF systems of breast tumor-bearing mice. (**A**) Dynamics of CTCs in blood circulation during tumor development measured by FFC; (**B**) PA scanning of the SLN; high-amplitude signals color-coded by yellow and red are associated with objects ≤15 μm; (**C**) Two-color (670 nm and 820 nm) PAFC of CSF *in vivo* through the skull.

Irradiation of CSF through a 300 µm-diameter fiber in the neck area of nude mice with nanosecond laser pulses at 671 nm revealed rare PA signals with a rate of a few signals per hour associated with targeted breast tumor cells flowing through the CSF (Figure 24C). At indicated above for the blood CTC rate, the total number of CTCs in CSF was estimated to be 4–6 CTCs in the entire CSF volume (~40 mL for a mouse). Examination of brain specimens *ex vivo* and *in vitro* by high-resolution optical microscopy and histology revealed that more than half of the mice did not have brain micro- or macrometastases as a potential source of CTCs, while all mice had blood CTCs at the average rate indicated above. Thus, pre-metastatic CTCs in CSF could have originated from blood CTCs. The estimated sensitivity was approximately a few CTCs among a million WBCs over a 4 h period. Our approach may provide a semi-automated PA molecular analysis that vastly improves the sensitivity, reliability, objectivity, and accuracy of detecting tumor cells in CSF as compared to CSF cytology. Due to high depth assessment (up to few centimeters), fiber-based PAFC could be applied to different sites of the spinal canal (e.g., cervical, lumbar) and ventricles by a procedure that is well established for lumbar puncture. If clinically successful, this tool may provide a tremendous leap forward in a previously unexplored scientific area, *in vivo* CSF cell testing, including cell trafficking, cell-to-cell interactions, and the circulation routes, especially in the terminal end of the spinal canal.

### 4.6. *In Vivo* PA Detection in Bone

Bone is one of the most common sites of metastasis in breast cancer patients. Although bone marrow micrometastasis is associated with poor survival, early detection is not yet a part of routine medical practice due to the limitation of current diagnostic techniques. *Ex vivo* examination of a biopsy specimen with conventional assays (e.g., microscopic, immunocytochemistry, RT-PCR) is an invasive, painful, time-consuming, discontinuous procedure, with very limited and discrete time points. Despite progress in diagnostic techniques *in vivo* (e.g., MRI, PET, CT, and optical assays), no clinically relevant method has been developed for the early detection of bone micrometastasis. As an alternative, PA imaging, based on the conversion of absorbed energy in acoustic waves in low-toxicity NPs, provides higher sensitivity and resolution more deeply in tissue (up to 5–7 cm) than most optical modalities. However, *in vivo* PA detection of bone metastasis has not yet been proposed. 

We hypothesized that the PAFC platform can be used for non-invasive detection of early breast cancer metastasis in bones. The method is based on the administration of strongly absorbing NPs functionalized to specific breast cancer cell markers that are typically not expressed in normal blood and bone marrow, followed by an *in vivo* PA examination of the bones of interest by exposing the skin above the specific bones to a NIR pulsed laser. Healthy bones produce a low level of background PA signals because there is a low concentration of NPs randomly and non-specifically distributed in the bone tissue. In contrast, a high localized concentration of NPs in a targeted bone metastasis provides PA signal enhancement above the background as evidence of disease progression. The key hypothesis is that micrometastses down to few cancer cells in bones can be targeted *in vivo* by functionalized gold NPs and then detected with high-sensitivity PAFC techniques. 

To test this hypothesis, we demonstrated (1) PA detection of mimic metastatic cells labeled by bioconjugated NPs *in vitro*, (2) detection of mimic metastatic breast cancer cells in the tibia (previously labeled *in vitro*) in a mouse model, (3) detection in the tibia of unlabeled mimic metastatic cells labeled with NPs, and 4) detection of real metastases in a mouse breast tumor model by labeling them with bioconjugated NPs *in vivo.* Briefly, the main experiments involved (1) the breast cancer cell line MDA-MB-231 positive for the folate marker; (2) ~10 × 35 nm GNRs with maximum absorption near 670 nm (GNR_670_) (Nanopartz, Inc.) functionalized against folate receptors; (3) nude mice *nu/nu* (Harlan Sprague-Dawley); (4) a PA setup using available high-pulse-rate lasers and an ultrasound transducer (unfocused XMS-310, 10 MHz, and focused V316-SM, 20 MHz, with amplifiers from Panametrics); and (5) the established protocols for labeling and animal bone metastasis. 

**Figure 25:**
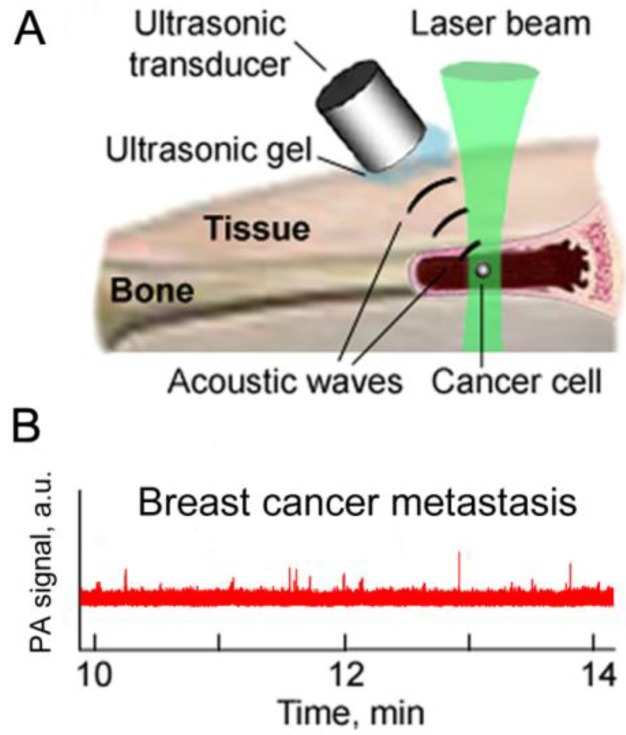
*In vivo* PA bone flow cytometry. (**A**) Schematics; (**B**) Non-invasive in vivo molecular targeted detection of breast CTCs in mouse tibia.

We determined that even after significant attention of laser radiation in the bone tissue, the laser energy is still enough to generate readable PA signals from strongly absorbing objects inside bones using focused ultrasound transducers (Figure 25A). Indeed, intravenous injection of a carbon nanotube (CNT) cluster as a rough cell phantom led to the appearance of PA signal traces from the mice’s tibia [92]. To exclude possible PA background signals from CNTs, circulating in blood vessels between fiber used for delivery laser radiation and bones, the fiber was gently attached to the skin in an area with no visible vessels. A time-resolved detection system was used to select PA signals coming with time delay from relatively deep bone as compared to the depth of superficial blood vessels. Scanning of the laser beam along the bone revealed rare stationary PA signals associated with the accumulated CNTs in bone. Then similar results were obtained for breast cancer cells (MDA-MD-321-GFP) targeted by GNRs with a maximum absorption at 670 nm [28]. The GNRs conjugated with folate were intravenously injected in the tumor–bearing mouse model at Week 4 after tumor inoculation. We observed rare PA signals from the tibia irradiated by a high-pulse-rate laser (10 kHz) at 671 nm (Figure 25B). These signals were associated with individually targeted CTCs. PA scanning cytometry revealed also rare stationary PA signals associated with the CTCs captured in bone. This technique, after further optimization, has the potential for early and painless diagnosis of bone cancer metastasis (non-invasive bone marrow biopsy) or infection through the administration of strongly absorbing NPs functionalized to identify specific cancer cells or infection markers. The identification of targeted cells could be performed in analogy to detection of micrometastasis in SLNs (see above). In particular, healthy bones produce low-level background PA signals because low concentrations of NPs are randomly and non-specifically distributed in the bone tissue as compared to a high local concentration of NPs that is found in cells, causing bone metastasis with possible red-shift effect in clustered plasmonic NPs. Eventually, we successfully demonstrated that the proposed *in vivo* non-invasive PA bone marrow biopsy has the potential to provide a breakthrough in the ultrasensitive detection of early (*i.e.*, still curable) metastasis, disease staging/progression, recurrence monitoring, and evaluation of therapy efficacy (through the decrease of PA signals). 

### 4.7. *In Vivo* Flow Cytometry with Bypass Schematics

**Figure 26:**
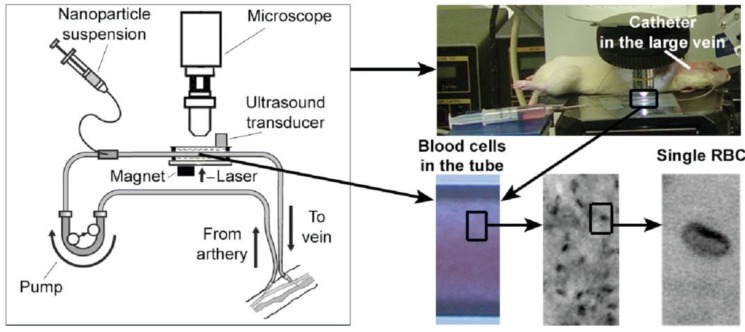
Principle and animal model for *in vivo* PA detection, molecular targeting, magnetic capturing and PT ablation of CTCs in blood with an extracorporeal schematic.

One of possible applications *in vivo* PAFC on human, especially using theranostic approach, can be based on the use of extracorporeal schematics [94]. We performed a preliminary study of this approach on animal models in relation to *in vivo* PA detection, molecular targeting, and PT purging of melanoma and breast CTCs (Figure 26). A catheter was placed in a large vein to create a bypass. MNPs, conjugated with Abs against melanoma markers (MSCP) were injected into a tube. The distance between the injection site and detection points could vary by changing the tube length. In this model, cells labeled in flow could be captured by the magnet. Laser irradiation of the area near the magnet generates PA signals, which can be detected with an ultrasound transducer attached to the tube. Simultaneously, laser irradiation at a higher energy can allow PT killing of targeted CTCs. Conventional transmission imaging made it possible to simultaneously control the positions of the laser beam, magnet, and transducer. In particular, high-speed imaging also allows visualization of individual moving cells at the single-cell level as shown in the images (right, bottom) obtained at different magnifications (4×, 20×, and 100×, respectively). Thus, the extracorporeal (bypass) schematic could provide continuous PA monitoring of blood flow in external tubes, and permit efficient capture of abnormal objects (e.g., CTCs) targeted by MNPs directly in the extracorporeal flow. Magnetic capture of both abnormal objects and unbound MNPs prevents them from being transported further in the systemic circulation.

### 4.8. *In Vitro* Flow Cytometry Integrating PA, PT and Fluorescence Detection Schematics

The verification of the unique data obtained with PAFC *in vivo* can be performed *ex vivo* at a relatively high CTC concentration, (>1–10 CTCs/mL) detectable with conventional CTC assays. As mentioned above, *in vitro* FC is a well-established analytical tool that provides multiparameter quantification of cancer cells using fluorescence and scattering cell or labels properties. However, modern FC is not applicable for analysis of nonfluorescent cells and labels. Ironically, FC was initially introduced as an absorption FC based on measurement of light attenuation [31,32]. However, due to low sensitivity related to a short optical pathway in single cells, this method was later replaced with fluorescent excitation/emission techniques, which requires fluorescent labeling. FC with PT and PA detection methods [88,97,107] can dramatically enhance the absorption sensitivity by 4–5 orders of magnitude which allow label-free detection of absorbing non-fluorescent cells and probes in flow. Moreover, PT and PA techniques provide detection and imaging single QDs with sensitivity comparable with that of fluorescence imaging (because most of the absorbed energy in QDs is converted into heat [112]) which makes the PAFC/PTFC platform even more compelling for a range of labeling strategies [28]. 

**Figure 27:**
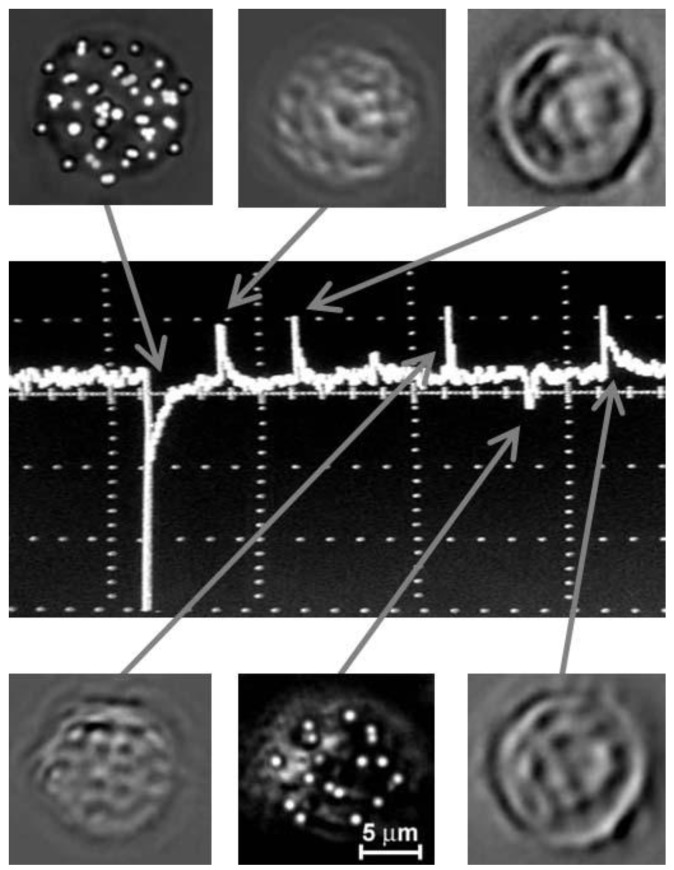
Middle, Tracings of the PT signals (time scale, 200 ms/div) from leukemia cells. Top and bottom, Corresponding PT images. OPO parameters: wavelength, 530 nm; pulse rate, 100 Hz; fluence, 100 mJ/cm^2^ [75].

PT imaging of mouse RBCs and WBCs and human cancer cells (leukemia, K562) (Figure 27) was performed using a syringe pump–driven system in 100-µm capillary flow tubes and OPO at 100 Hz [75]. The specific intrinsic PT image structures obtained from cancer cells could be used for their identification. Moreover, cell labeling with gold NPs may significantly enhance both PT signal amplitude and PT image contrast. The positive peaks indicate a standard linear PT signal from single cells without NP enhancement (Figure 27, middle). Because of the short duration of each PT signal (15–25 µs), the signals are compressed at slow signal tracing. The presence of strong negative peaks in NP-bound cells (one to two orders of magnitude greater than that of a cell with no NP binding) in the PT signals was due to the formation of nanobubbles around overheated NPs due to their extremely high localized absorption compared to intrinsic cellular absorption. Other pilot studies performed with simultaneous monitoring of PT images has confirmed this finding: PT images of cells tagged with NP-conjugated antibodies (Figure 27, top left and bottom center) revealed highly localized, NP-associated bright spots (Figure 27, top, left, bottom, middle) as compared to low contrast of intrinsic cytochrome-related structures (Figure 27, top, center and right). 

Recently, we have developed a novel PAFC modules which were operated with relatively large customized flow tube (up to 0.8–2 mm) [35,75] or incorporated into conventional *in vitro* FC schematics [87,90,100]. The integrated system allows simultaneous measurements of light absorbance, scattering, and of multicolor fluorescence from single cells in the flow at rates up to 2 m/s. We compared various combinations of excitation laser sources for multicolor detection, including simultaneous excitation of PA and fluorescence using a single 500 kHz pulsed nanosecond laser. The multispectral detection scheme allows for simultaneous detection of various fluorescent and absorbing PA contrast agents. These new techniques were used for studies of NP uptake and for the analysis of cell line pigmentation, including genetically encoded melanin expression in breast cancer cells [63].

**Figure 28:**
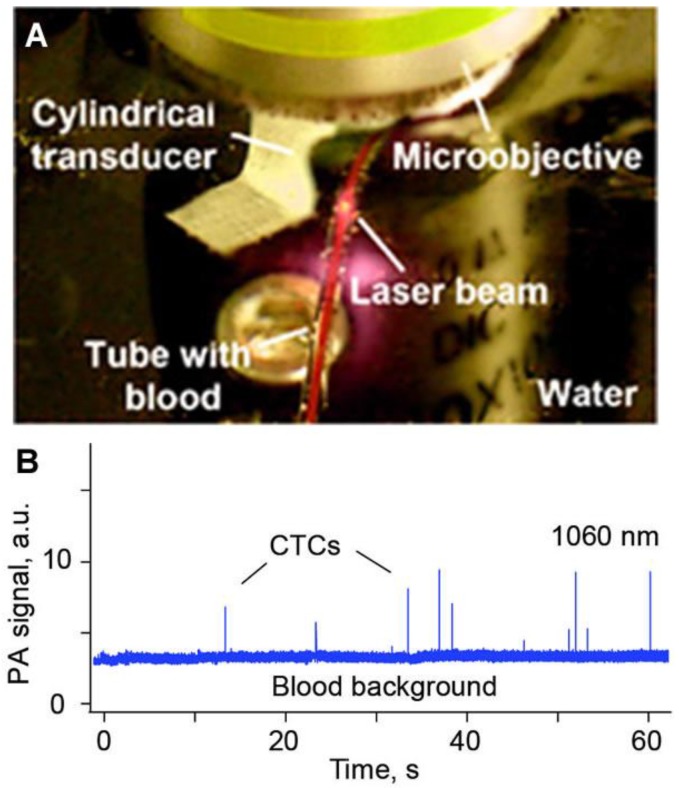
(**A**) *In vitro* PAFC with focused cylindrical transducer; (**B**) Typical PA signal trace from rare melanoma cells (B16F01) in 0.8 mm flow tube with mouse blood extracted from tumor-bearing mouse model [82]. Laser parameters: wavelength, 1,064 nm; pulse width 10 ns; pulse rate, 10 kHz; pulse energy, up 50 µJ; linear beam size, 5 µm × 1 mm. Customized ultrasound transducer parameters: type, focused cylindrical; frequency, 32 MHz; focal length, 8 mm; lateral resolution, 45 µm.

In particular, with in vitro PAFC schematics shown in Figure 28A we obtained PA signals from single melanoma CTCs in unprocessed blood sample (1.5–2 mL) collected from a melanoma (B16F10)-bearing mouse. Melanin in this study was used as an intrinsic PA contrast agents [82]. 

For some the e*x vivo* studies, we used a flow module consisting of a 0.8 mm glass tube with a flow velocity of 1 and 35 cm/s, respectively (*i.e.*, rate in the range of 0.3–10 mL/min) and with NIR lasers (1,064 nm) with linear beams of 5 µm × 1 mm. PA signals were detected with a cylindrical ultrasound transducer located in a water bolus around the tube. Calibration of the PAFC *in vitro* with blood from healthy mice (*i.e.*, no tumor) revealed no transient PA signals. In blood sample with CTCs PAFC revealed a larger (2–3-fold) number of CTC-associated PA signals, respectively in whole blood than in samples in which RBCs were partly removed. These data confirm that PAFC *in vitro* requiring no sample processing is faster (10–100-fold) and more accurate than existing CTC assays *in vitro* involving multistep processing. Thus, *in vitro* PAFC can be used to verify PA data *in vivo* when concentrations of CTCs are high enough to be detected *in vitro* and even independently for CTC detection *in vitro.* Based on obtained data using unprocessed mouse blood with melanoma CTCs, we estimated that this technology can provide *in vitro* rapid monitoring of unprocessed human blood sample with volume of 40 mL during just a 5–10 min with sensitivity threshold of a 1 CTC/40 mL that impossible with existing CTCs assays with current sensitivity of 1 CTC/mL [1,2,3,4,5,6,7,8,9,10,11,12,13,14,15,16,17,18,113,114,115,116]. Moreover, we demonstrated magnetic trapping of cells labeled with MNPs in flow *in vivo* and *in vitro* using integrated fiber-magnetic-PA probes [81] that can increase PAFC sensitivity to 100-fold by increase local concentration of trapped CTCs (Figure 18B) and/or by averaging more PA signals from nonmoving trapped CTCs, hypothetically from one CTC. 

## 5. Conclusions

In this review, we have summarized recent advances in the development of a new concept of *in vivo* blood cancer test using the principle of *in vivo* flow cytometery with PA detection schematics (PAFC). This biomedical tool using label-free detection of pigmented melanoma CTCs or molecular targeting of non-absorbing CTCs (e.g., breast cancer) with functionalized plasmonic NPs and preclinical animal models can provide the insight on metastasis development and CTC distribution in blood, lymph, bone and cerebrospinal fluids. We revealed that some diagnostic or therapeutic procedures (e.g., palpation, biopsy, or surgery) can enhance CTC release from primary tumors in circulation, increasing the risk of metastasis. The presented results suggest the excellent potential of PAFC and its combination with PT and fluorescence methods as a new promising biotechnical platform in biological research. The ability to assess a large blood volume *in vivo* [potentially the patient’s entire blood volume (in adults ~5 L)] significantly (10^3^-fold) may enhance the sensitivity of CTC detection including rare cancer stem cells compared to the existing CTC assay *ex vivo.*

Translation of *in vivo* FC to human faces many challenges such as: toxicity of probes, strong scattering, absorption, and an autofluorescence background, laser safety issue and assessment deep large vessels. Nevertheless, the clinical significance and safety of PA devices have already been demonstrated in many clinical trials, including the monitoring of cerebral blood oxygenation in ~1 cm-diameter human jugular veins at a depth of 1–2 cm and imaging of breast tumors, melanoma, and sentinel lymph nodes. We recently developed a portable clinical prototype of PAFC using a high-pulse-repetition rate laser at 1,064 nm with high pulse rate and a focused, ultrasound transducer gently attached to the skin near selected blood vessels. Our clinical goal is to detect CTCs in hand vessels with diameters of 1–1.5 mm at 1–2 mm depths (easily accessible with PAFC with depth of 3–5 cm and 5–7 cm in a single study). 

Despite missing some weakly pigmented cells, we expect that label-free PAFC technology may have high clinical significance for early melanoma diagnosis, and melanoma recurrence because in these cases, the fact that CTCs are present in blood flow may have higher importance than accurate CTCs count. In the future, the problem of false-negativity in label-free PAFC could be addressed via molecular CTCs targeting by conjugated low-toxicity NPs. If oncoming pilot clinical trials using the portable PAFC device are successful, this technology can provide breakthroughs for the early detection of CTCs when metastasis has not yet developed and, hence well timed therapy including PT therapy is more effective. Taking into account recently developed by us super-resolution PT and PA microscopy with resolution beyond the diffraction and spectral limits [64,103] we expect that similar approach can be used for improvement the resolution of nonlinear OR-PAFC using tunable laser with strongly focused optical beams. In particular, it will allow to distinguish individual CTC in CTC aggregates. 
